# K_v_1.1 preserves the neural stem cell pool and facilitates neuron maturation during adult hippocampal neurogenesis

**DOI:** 10.1073/pnas.2118240119

**Published:** 2022-05-25

**Authors:** Yuan-Hung Lin King, Chao Chen, John V. Lin King, Jeffrey Simms, Edward Glasscock, Shi-Bing Yang, Yuh-Nung Jan, Lily Y. Jan

**Affiliations:** ^a^Neuroscience Graduate Program, University of California, San Francisco, CA 94143;; ^b^Department of Physiology, University of California, San Francisco, CA 94143;; ^c^Department of Biochemistry and Biophysics, University of California, San Francisco, CA 94143;; ^d^HHMI, University of California, San Francisco, CA 94143;; ^e^Behavioral Core, Gladstone Institute of Neurological Disease, Gladstone Institutes, San Francisco, CA 94158;; ^f^Department of Biological Sciences, Southern Methodist University, Dallas, TX 75275;; ^g^Institute of Biomedical Sciences, Academia Sinica, Taipei, 115, Taiwan;; ^h^Neuroscience Program of Academia Sinica, Academia Sinica, Taipei, 115, Taiwan

**Keywords:** voltage-gated potassium channel, adult neurogenesis, hippocampus, learning and memory

## Abstract

Adult hippocampal neurogenesis is critical for learning and memory, and aberrant adult neurogenesis has been implicated in cognitive decline associated with aging and neurological diseases [J. T. Gonçalves, S. T. Schafer, F. H. Gage, *Cell* 167, 897–914 (2016)]. In previous studies, we observed that the delayed-rectifier voltage-gated potassium channel K_v_1.1 controls the membrane potential of neural stem and progenitor cells and acts as a brake on neurogenesis during neonatal hippocampal development [S. M. Chou *et al.*, *eLife* 10, e58779 (2021)]. To assess the role of K_v_1.1 in adult hippocampal neurogenesis, we developed an inducible conditional knockout mouse to specifically remove K_v_1.1 from adult neural stem cells via tamoxifen administration. We determined that K_v_1.1 deletion in adult neural stem cells causes overproliferation and depletion of radial glia-like neural stem cells, prevents proper adult-born granule cell maturation and integration into the dentate gyrus, and moderately impairs hippocampus-dependent contextual fear learning and memory. Taken together, these findings support a critical role for this voltage-gated ion channel in adult neurogenesis.

## Significance

Despite decades of research on adult neurogenesis, little is known about the role of bioelectric signaling in this process. In this study, we describe how a voltage-gated potassium channel, K_v_1.1, supports adult neurogenesis by maintaining the neural stem cell niche and facilitating newborn neuron development. Additionally, we show that deletion of K_v_1.1 from adult neural stem cells contributes to modest impairments in hippocampus-dependent contextual fear learning and memory. Dysfunctional adult neurogenesis has been implicated in cognitive decline associated with aging and neurological disease. Therefore, understanding the role of K_v_1.1 in adult neurogenesis represents an opportunity to identify new therapeutic targets to promote healthy neurogenesis and cognition.

The subgranular zone of the hippocampus is one of two well-characterized neurogenic niches in the adult mouse brain. Integration of adult-born granule cells into the dentate gyrus is important for learning and memory, and impaired adult neurogenesis has been implicated in neurodegenerative and neuropsychiatric diseases (1–6). Adult hippocampal neurogenesis is divided into several developmental stages. Initially, quiescent neural stem cells with radial glia-like morphology—known as radial glia-like neural stem cells (type 1 cells)—activate and proliferate, either self-renewing or differentiating into intermediate neural progenitor cells with a glia-like phenotype (type 2a cells). As these cells differentiate, they lose their stem cell properties and display more neuron-like features (type 2b cells). Then, they develop into neuroblasts (type 3 cells) within a few days. Over the course of the next 2 wk to 4 wk, type 3 cells give rise to immature adult-born neurons that extend an apical dendrite into the dentate granule cell layer and grow secondary and tertiary dendrites. Simultaneously, they migrate from the subgranular zone into the dentate granule cell layer. Finally, adult-born granule cells mature into highly excitable neurons and integrate into the dentate gyrus circuitry (1–8).

While adult hippocampal neurogenesis is regulated by various environmental and endogenous factors, recent studies have also begun to explore the role of bioelectric signaling in this process. In nonexcitable cells, such as neural stem and progenitor cells, changes in the membrane potential can orchestrate proliferation, differentiation, migration, and survival during development (9). The membrane potential is controlled by ion channels, and ion channel dysfunction often results in neurodevelopmental disorders (10, 11). Interestingly, ion channels continue to modulate the membrane potential and cell dynamics of neural stem and progenitor cells during postnatal neurogenesis (12–19). In adult radial glia-like neural stem cells, gap junctions and inward rectifying potassium channels maintain the membrane potential (14, 17, 19). Their proliferation is also regulated by GABAergic and glutamatergic signaling, where these neurotransmitters activate their corresponding ligand-gated ion channels to alter the membrane potential (13, 15, 18). Additionally, local circuit activity is critical for young adult-born granule cells, which receive, in order, depolarizing GABAergic inputs, excitatory glutamatergic inputs, and, finally, inhibitory GABAergic inputs to advance through stages of maturation and survival (20–23).

In this study, we examine the role of the voltage-gated potassium channel K_v_1.1 in adult hippocampal neurogenesis. K_v_1.1 is encoded by the *Kcna1* gene in mice, and its expression begins increasing at ∼2 wk after birth and stabilizes in adulthood (24). K_v_1.1 is well known for its role in regulating neuronal excitability and seizure activity (25, 26). Mice without functional K_v_1.1—K_v_1.1 null mutant mice and *megencephaly* (*mceph*) mice—not only develop seizures but also have an abnormally increased number of neurons in the dentate gyrus (12, 27–32). Using mosaic analysis with double markers (MADM) (33–35) in heterozygous *mceph* mice, we showed that K_v_1.1 regulates neurogenesis in a cell-autonomous manner (32). We also found that loss of K_v_1.1 function in K_v_1.1 null mice depolarizes neonatal neural progenitor cells and increases proliferation through enhanced TrkB signaling (12). Because K_v_1.1 null mice exhibit seizures beginning a few weeks after birth (27, 29–31), and seizure activity can affect neurogenesis (36, 37), it has not been feasible to assess the function of K_v_1.1 in adult neurogenesis.

To address this issue and clarify the role of K_v_1.1 in adult hippocampal neurogenesis, we created inducible K_v_1.1 conditional knockout (K_v_1.1 cKO) mice, which allowed us to specifically delete *Kcna1* from adult neural stem cells via tamoxifen injection and eliminate the confounding effect of seizures in our study. Using this mouse model, we first removed K_v_1.1 in neonatal neural stem cells to validate our previous results with improved temporal resolution. Indeed, we recapitulated our previous observations showing that loss of K_v_1.1 in neonatal neural stem cells increases proliferation and neuron production. Interestingly, the role of K_v_1.1 in adult neural stem cells is more complex. We discovered that K_v_1.1 prevents overproliferation and depletion of radial glia-like neural stem cells and enables proper adult-born granule cell maturation and positioning during adult neurogenesis. We further corroborated our findings of an age-dependent role of K_v_1.1 using MADM of heterozygous K_v_1.1 (*Kcna1^+/−^*) mice (33–35). Finally, we determined that decreased adult neurogenesis in K_v_1.1 cKO mice causes deficits in hippocampus-dependent contextual fear conditioning. These results demonstrate that K_v_1.1 expression in adult neural stem cells is integral for preserving hippocampal neurogenesis and contextual learning and memory.

## Results

### Time-Controlled Deletion of K_v_1.1 from Neural Stem Cells.

To investigate the function of K_v_1.1 in neural stem cells at various postnatal stages, we generated K_v_1.1 cKO mice. We bred mice expressing a tamoxifen-inducible Cre recombinase (Cre) in neural stem cells (Nestin-Cre^ERT2^) (38–40) with *Kcna1* floxed mice (*Kcna1^fl/fl^*) (41) and Cre reporter mice (PC::G5-tdT) (42) to achieve temporal and cell type–specific control of *Kcna1* deletion. Upon tamoxifen injection, Cre begins expressing in neural stem cells, resulting in the removal of K_v_1.1 and expression of tdTomato and GCaMP5G in the neural stem cells and their progeny. While the Cre-expressing neural stem cells are a small subset of all cell types in the dentate gyrus, the expression of tdTomato and GCaMP5G in these cells enables us to identify them for lineage tracing. We amplified the GCaMP5G signal with an anti-GFP antibody, because the anti-GFP antibody was compatible with our histology techniques using multiple cell markers. In this way, we successfully read out Cre expression in neural stem cells. To control for the possible effects of tamoxifen, Cre, and reporter expression on neural stem cell dynamics, we bred mice with wild-type *Kcna1* (*Kcna1^+/+^*) with Nestin-Cre^ERT2^ and PC::G5-tdT mice for our control cohort (K_v_1.1 WT) (*SI Appendix*, Fig. S1*A*).

To validate K_v_1.1 knock-out after tamoxifen injection, we injected 8-wk-old K_v_1.1 cKO and K_v_1.1 WT mice with tamoxifen for three consecutive days and used fluorescence-activated cell sorting to isolate Cre-expressing tdTomato+ cells from the dentate gyrus at 2 wk post tamoxifen injection (*SI Appendix*, Fig. S1 *B*–*E*). We found that *Kcna1* messenger RNA expression was decreased by ∼90% in tdTomato+ cells in the dentate gyrus of K_v_1.1 cKO mice compared to those of K_v_1.1 WT mice (*P* = 0.0001) (*SI Appendix*, Fig. S1*F*). We also recorded the resting membrane potential of acutely dissociated tdTomato+ cells from the dentate gyrus at 2 wk post tamoxifen injection to determine whether K_v_1.1 has been functionally deleted (*SI Appendix*, Fig. S2*A*). To assess the effect of acute K_v_1.1 inhibition on resting membrane potential, we applied the selective K_v_1.1 blocker, Dendrotoxin-K (DTx-K), to tdTomato+ K_v_1.1 WT cells and observed a depolarized resting membrane potential (−70 ± 2.6 mV) compared to untreated K_v_1.1 WT cells (−87 ± 0.58 mV) (*P* = 0.0002) (*SI Appendix*, Fig. S2 *B* and *C*). Consistent with our findings using DTx-K for acute K_v_1.1 inhibition, we found that the resting membrane potential of tdTomato+ K_v_1.1 cKO cells was similarly depolarized (−68 ± 1.0 mV) compared to K_v_1.1 WT cells (*P* = 0.0002). Together, these results show that K_v_1.1 is functionally knocked out of the Cre-expressing tdTomato+ neural stem cell lineage of K_v_1.1 cKO mice by 2 wk post tamoxifen injection, likely resulting in depolarized cells.

### Conditional Knockout of K_v_1.1 in Neonatal Neural Stem Cells Increases Early Postnatal Hippocampal Neurogenesis.

Both K_v_1.1 null mice and *mceph* mutant mice display increased neonatal neurogenesis before seizure onset around 1 mo after birth (12, 27–32). As K_v_1.1 cKO mice allowed us to examine the role of K_v_1.1 in early postnatal neurogenesis with more precise temporal resolution, we focused on the role of K_v_1.1 during peak hippocampal development at postnatal day 7 (P7), just before the second postnatal week when hippocampal neurogenesis begins transitioning from a more embryonic stage to adult stage (43, 44).

We injected tamoxifen at P0 to knock out K_v_1.1 and conducted lineage tracing by injecting Bromodeoxyuridine (BrdU), which is incorporated into the DNA of actively dividing cells (45–48), at P7. We then quantified the number of progeny cells in the dentate gyrus of K_v_1.1 cKO mice and K_v_1.1 WT mice at P14 (*SI Appendix*, Fig. S3*A*). To determine which cell types were altered in the Cre-expressing GFP+ neural stem cell lineage, we costained the sections with established neural stem and progenitor cell marker, Sox2, and the postmitotic neuronal marker, NeuN. We found that neural stem and progenitor cell progenies from cells dividing at P7 (GFP+, BrdU+, Sox2+) in K_v_1.1 cKO subgranular zone were increased by ∼60% (*P* = 0.036) (*SI Appendix*, Fig. S3 *B* and *C*). Within the dentate granule cell layer, neurons produced from cells dividing at P7 (GFP+, BrdU+, NeuN+) were increased by ∼55% (*P* = 0.0007) (*SI Appendix*, Fig. S3 *D* and *E*). The enhanced neonatal neurogenesis that we observed in K_v_1.1 cKO mice is similar to our previous findings in K_v_1.1 null mice (12), providing further evidence that K_v_1.1 acts as a brake on early postnatal neurogenesis.

### Deletion of K_v_1.1 in Adult Neural Stem Cells Leads to a Transient Activation Followed by a Depletion of Radial Glia-Like Cells.

Next, we investigated the role of K_v_1.1 in adult hippocampal neurogenesis. To specifically ablate K_v_1.1 in the adult neural stem cell lineage, we injected 8-wk-old adult mice with tamoxifen for three consecutive days. Unlike the K_v_1.1 null mice and *mceph* mutant mice, adult K_v_1.1 cKO mice injected with tamoxifen did not display seizure phenotypes, thereby allowing us to eliminate the confounding effects of seizures on adult neurogenesis from our study.

**Fig. 1.**
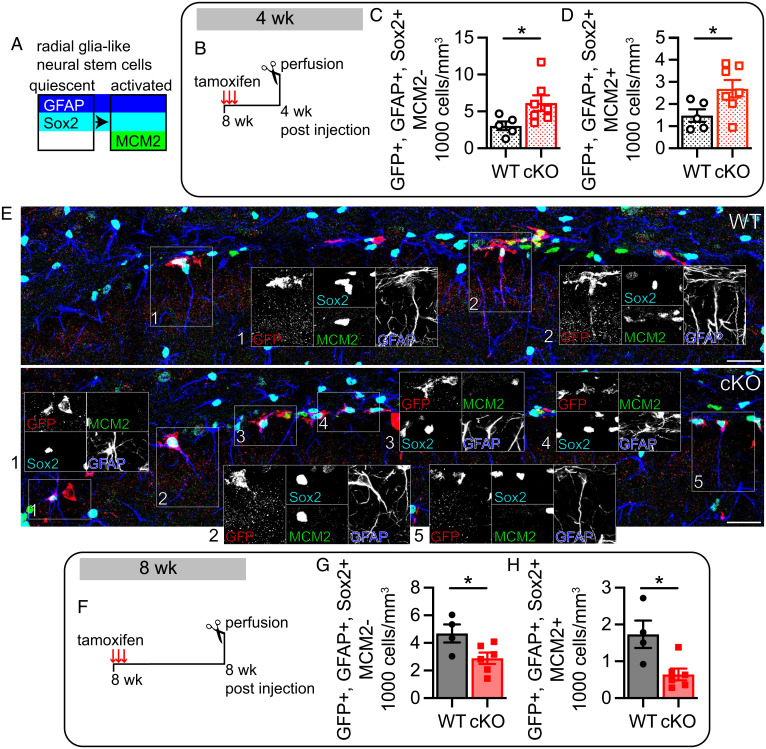
Deletion of K_v_1.1 in adult neural stem cells results in an initial increase of radial glia-like neural stem cells before eventual depletion of the radial glia-like neural stem cell pool. (*A*) Diagram of cell marker expression during neural stem cell development. Quiescent radial glia-like neural stem cells express GFAP and Sox2. As radial glia-like neural stem cells start proliferating, they express MCM2. (*B*) Protocol to assess short-term effects of K_v_1.1 cKO on adult hippocampal neurogenesis. At 8 wk old, K_v_1.1 cKO mice and K_v_1.1 WT mice were injected with tamoxifen for three consecutive days to induce Cre expression and, in K_v_1.1 cKO mice, *Kcna1* deletion. At 4 wk post tamoxifen injection, we carried out immunostaining of GFP+ radial glia-like neural stem cells. (*C* and *D*) Quantification (cells per cubic millimeter) of adult radial glia-like neural stem cells at 4 wk post tamoxifen injection. Quiescent radial glia-like neural stem cells (GFP+, GFAP+, Sox2+, MCM2−) (*P* = 0.048) and activated radial glia-like neural stem cells (GFP+, GFAP+, Sox2+, MCM2+) (*P* = 0.046) were increased in K_v_1.1 cKO mice (*n* = 7) compared to K_v_1.1 WT mice (*n* = 5). (*E*) Representative image showing expression of GFP (red), GFAP (blue), Sox2 (cyan), and MCM2 (green) in the ventral blade of the dentate gyrus in K_v_1.1 WT mice (*Top*) and K_v_1.1 cKO mice (*Bottom*). Within the K_v_1.1 WT overlay, quiescent (2) and activated (1) radial glia-like neural stem cells are boxed; within the K_v_1.1 cKO overlay, quiescent (1, 3 to 5) and activated (2) radial glia-like neural stem cells are boxed. Each individual channel of the boxed areas is displayed. (Scale bar, 25 µm.) (*F*) Protocol to assess long-term effects of K_v_1.1 cKO on adult neurogenesis. K_v_1.1 cKO mice and K_v_1.1 WT mice were injected at 8 wk of age with tamoxifen for three consecutive days to induce Cre expression and *Kcna1* deletion. At 8 wk post tamoxifen injection, we carried out immunostaining of GFP+ radial glia-like neural stem cells. (*G* and *H*) Quantification (cells per cubic millimeter) of adult radial glia-like neural stem cells at 8 wk post tamoxifen injection. Quiescent radial glia-like neural stem cells (GFP+, GFAP+, Sox2+, MCM2−) (*P* = 0.039) and activated radial glia-like neural stem cells (GFP+, GFAP+, Sox2+, MCM2+) (*P* = 0.016) were decreased in K_v_1.1 cKO mice (*n* = 6) compared to K_v_1.1 WT mice (*n* = 4). (*C*, *D*, *G*, and *H*) Unpaired two-tailed Student’s *t* test. *P < 0.05. Data are presented as mean ± SEM.

We started by examining mice 4 wk after tamoxifen injection to assess the early effects of K_v_1.1 deletion on adult quiescent radial glia-like neural stem cells (type 1 cells), which are labeled by Sox2 and the glial marker, GFAP. As they become activated, radial glia-like neural stem cells begin expressing the mitotic marker, MCM2 (Fig. 1*A*) (49). At 4 wk post tamoxifen injection, quiescent (GFP+, GFAP+, Sox2+, MCM2−) and activated (GFP+, GFAP+, Sox2+, MCM2+) radial glia-like neural stem cells were increased by ∼100% (*P* = 0.048) and ∼80% (*P* = 0.046), respectively, in K_v_1.1 cKO mice as compared to K_v_1.1 WT mice (Fig. 1 *B–E*). This suggests that loss of K_v_1.1 initially promotes both radial glia-like neural stem cell division and self-renewal. Instead of self-renewing, radial glia-like neural stem cells can also divide and differentiate into type 2a cells, losing their GFAP expression (*SI Appendix*, Fig. S4*A*). To determine whether loss of K_v_1.1 alters radial glia-like neural stem cell differentiation, we quantified the amount of type 2a cells (GFP+, GFAP−, Sox2+, MCM2+) and found a trend toward statistical significance that type 2a cells in K_v_1.1 cKO mice were increased by ∼70% at 4 wk post tamoxifen injection (*P* = 0.068) (*SI Appendix*, Fig. S4 *B* and *D*). It is possible that the trend toward an increase of type 2a cells arose from either enhanced radial glia-like neural stem cell proliferation, which pushed radial glia-like neural stem cells to both self-renew and differentiate, or increased proliferation of both radial glia-like neural stem cells and type 2a cells in K_v_1.1 cKO mice. Interestingly, the increase in radial glia-like neural stem cells and type 2a cells did not lead to additional type 2b and proliferating type 3 cells (GFP+, GFAP−, Sox2−, MCM2+) (*SI Appendix*, Fig. S4 *C* and *D*). From these observations, K_v_1.1 expression seems to discourage adult radial glia-like neural stem cell division.

To determine the long-term effects of K_v_1.1 deletion, we investigated changes in the neural stem cell lineage at 8 wk after tamoxifen injection. Surprisingly, quiescent (GFP+, GFAP+, Sox2+, MCM2−) and activated (GFP+, GFAP+, Sox2+, MCM2+) radial glia-like neural stem cells were reduced by ∼40% (*P* = 0.039) and ∼65% (*P* = 0.016), respectively, in K_v_1.1 cKO mice as compared to K_v_1.1 WT mice (Fig. 1 *F–H*). We interpret this to mean that the initial increase in radial glia-like neural stem cell proliferation eventually exhausted their ability to self-renew and depleted the radial glia-like neural stem cell pool. We also examined the role of K_v_1.1 in type 2a and proliferating type 2b/3 cells. Because of the variability of the type 2a (GFP+, GFAP−, Sox2+, MCM2+) cell counts, we were unable to determine, with confidence, whether they were altered in the K_v_1.1 cKO mice at 8 wk post tamoxifen injection (*P* = 0.14) (*SI Appendix*, Fig. S4 *E* and *G*). We did not find a difference in type 2b/3 cells (GFP+, GFAP−, Sox2−, MCM2+) between K_v_1.1 cKO mice and K_v_1.1 WT mice at 8 wk post tamoxifen injection (*SI Appendix*, Fig. S4 *F* and *G*). Taken together, these results indicate that K_v_1.1 acts as a brake on overproliferation to prevent early depletion of the neurogenic stem cell pool.

### Eliminating K_v_1.1 from Adult Neural Stem Cells Prevents Proper Adult-Born Granule Cell Maturation and Positioning.

**Fig. 2.**
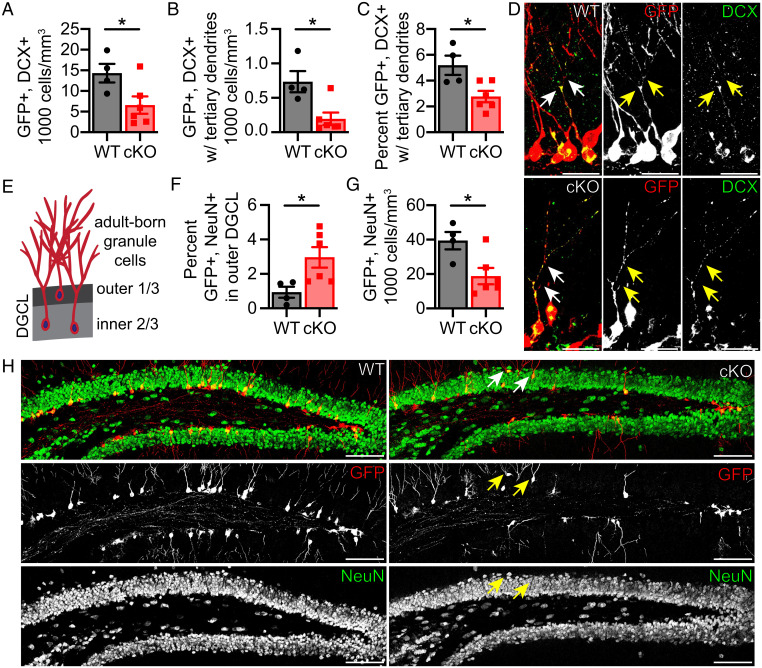
Loss of K_v_1.1 impairs adult hippocampal neurogenesis by altering doublecortin-expressing (DCX+) cell maturation and adult-born granule cell positioning. (*A*) Quantification (cells per cubic millimeter) of GFP+, DCX+ cells. Fewer GFP+, DCX+ cells were found in K_v_1.1 cKO mice (*n* = 6) compared to K_v_1.1 WT mice (*n* = 4) (*P* = 0.041). (*B*) Quantification (cells per cubic millimeter) of more-mature GFP+, DCX+ cells with tertiary dendrites. A decrease in GFP+, DCX+ cells with tertiary dendrites in K_v_1.1 cKO mice compared to K_v_1.1 WT mice was observed (*P* = 0.012). (*C*) Percentage of GFP+, DCX+ cells that display more-mature tertiary dendrite morphology was reduced in K_v_1.1 cKO mice compared to K_v_1.1 WT mice (*P* = 0.017). (*D*) Representative image of merged and individual signals of GFP (red) and DCX (green) in the dentate gyrus of K_v_1.1 WT mice (*Top*) and K_v_1.1 cKO mice (*Bottom*). Branching of DCX+ cells with tertiary dendrites is marked (arrows). (Scale bar, 25 µm.) (*E*) Cartoon of adult-born granule cell development. Adult-born granule cells migrate away from the subgranular zone toward the molecular layer and usually position themselves within the inner two-thirds of the dentate granule cell layer (DGCL). (*F*) Percentage of GFP+, NeuN+ cells in outer layer of the DGCL was diminished in K_v_1.1 cKO mice compared to K_v_1.1 WT mice (*P* = 0.033). (*G*) Quantification (cells per cubic millimeter) of GFP+, NeuN+ cells. GFP+, NeuN+ cells were decreased in K_v_1.1 cKO mice compared to K_v_1.1 WT mice (*P* = 0.020). (*H*) Representative image of overlayed and individual GFP (red) and NeuN (green) signals of the dentate gyrus from K_v_1.1 WT mice (*Left*) and K_v_1.1 cKO mice (*Right*) are shown. GFP+, NeuN+ cells in the outer third of the dentate granule cell layer are marked (arrows). (Scale bar, 100 µm.) (*A*, *B*, *C*, *F*, and *G*) Unpaired two-tailed Student’s *t* test; *P < 0.05. Data are presented as mean ± SEM.

To investigate the role of K_v_1.1 in later stages of adult-born granule cell production, we stained for a neurogenesis marker, doublecortin (DCX) at 8 wk post tamoxifen injection. DCX begins to express in a subset of type 2b cells and ceases to express as they become NeuN+ mature neurons (50–52). Interestingly, there was a ∼55% decrease in GFP+, DCX+ cells (*P* = 0.041) (Fig. 2 *A* and *D*) in K_v_1.1 cKO mice although the amount of proliferating type 2b/3 cells was not altered (*SI Appendix*, Fig. S4*F*). This raises the question whether the observed decrease in GFP+, DCX+ cells was due to the altered development of young adult-born granule cells. We relied on the distinct morphology of DCX+ cells at different stages of maturation to identify more-developed DCX+ adult-born granule cells as those with tertiary dendrites (50–52). Interestingly, in the K_v_1.1 cKO lineage, there was a ∼75% decrease in the number of GFP+, DCX+ cells with tertiary dendrites (*P* = 0.012) as well as a ∼45% decrease in the proportion of more-developed GFP+, DCX+ cells with tertiary dendrites among all GFP+, DCX+ cells (*P* = 0.017) (Fig. 2 *B–D*). Together, these results indicate that loss of K_v_1.1 hinders young adult-born granule cell maturation.

Those K_v_1.1 cKO neurons that successfully matured were also more likely to be inappropriately positioned. As young adult-born granule cells mature, they migrate from the subgranular zone into the dentate granule cell layer such that a majority are positioned within the inner two-thirds of the dentate granule cell layer (Fig. 2*E*) (20, 53). The percentage of mature GFP+ adult-born granule cells (GFP+, NeuN+) found in the outer third of the dentate granule cell layer at 8 wk post tamoxifen injection in K_v_1.1 cKO mice was ∼215% higher than that of K_v_1.1 WT mice (*P* = 0.033) (Fig. 2 *F* and *H*), indicating that loss of K_v_1.1 impairs adult-born granule cell positioning. These findings point toward a critical role of K_v_1.1 in facilitating successful adult-born granule cell development, as aberrant migration and positioning of adult-born granule cells has been found in mouse models of traumatic brain injury, schizophrenia, and neurodegeneration (54–56). These findings may also explain why we observed a ∼50% reduction in mature GFP+ adult-born granule cells (GFP+, NeuN+) in K_v_1.1 cKO mice by 8 wk post tamoxifen injection (*P* = 0.020) (Fig. 2 *G* and *H*). As young adult-born granule cells from neural stem cells lacking K_v_1.1 cannot properly mature and position themselves, they are likely unable to successfully integrate into the dentate gyrus circuitry. Taken together, our observations indicate that K_v_1.1 is integral for adult-born granule cells to develop proper morphology and positioning, which would allow them to incorporate synaptic inputs, integrate into the hippocampal circuitry, and fulfill their critical functions in learning and memory.

### MADM Analyses Reveal a Transient Increase of Neural Stem Cell Lineage Lacking K_v_1.1.

**Fig. 3.**
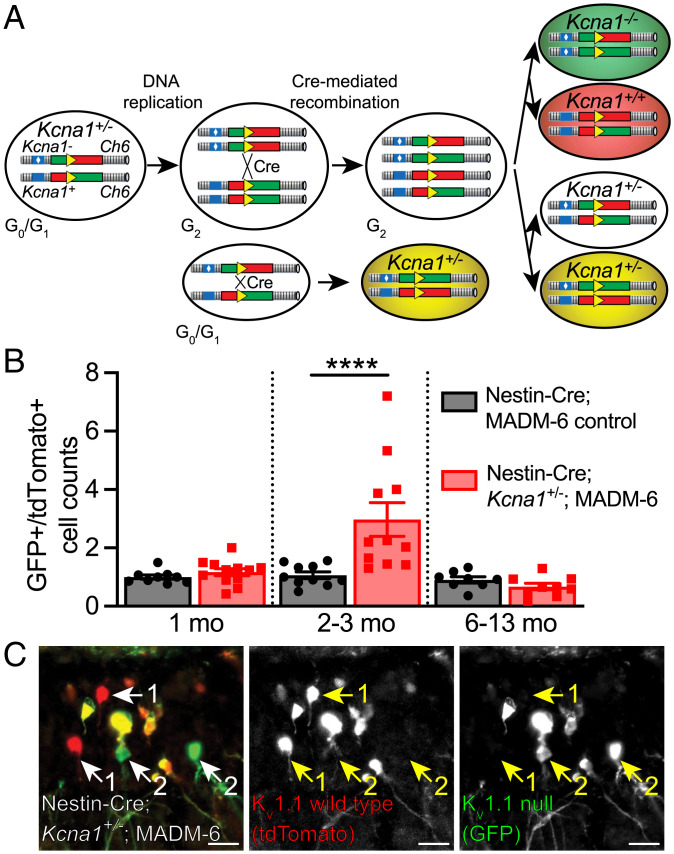
MADM analysis reveals a transient increase in K_v_1.1 null progeny cells in the dentate gyrus of 2- to 3-mo-old mice. (*A*) Schematic of MADM method (33–35) for generating K_v_1.1 null (GFP+) and K_v_1.1 wild-type (tdTomato+) progeny. MADM-6 marker mice were bred to *Kcna1*^+/−^ mice and Nestin-Cre mice. At G_2_ phase of the cell cycle, Cre induces infrequent interchromosomal recombination and restores functional GFP and tdTomato genes. During chromosome segregation, equal numbers of either *Kcna1*^−/−^ (GFP+) and *Kcna1*^+/+^ (tdTomato+) progenies or colorless *Kcna1*^+/−^ and dual-color *Kcna1*^+/−^ (yellow) progenies are generated. Dual-color *Kcna1*^+/−^ (yellow) progenies can also be generated by interchromosomal recombination at G_0_/G_1_ phase of the cell cycle (*Below*). (*B*) Ratio of GFP+ to tdTomato+ cells in Nestin-Cre;*Kcna1^+/−^*;MADM-6 and Nestin-Cre;MADM-6 control mice at 1 mo old, 2 mo to 3 mo old, and 6 mo to 13 mo old. The ratio of K_v_1.1 null (GFP+) to K_v_1.1 wild-type (tdTomato+) progeny cells was increased in 2- to 3-mo-old Nestin-Cre;*Kcna1^+/−^*;MADM-6 mice compared to Nestin-Cre;MADM-6 control mice (*P* < 0.0001). There was no difference in the ratio of K_v_1.1 null (GFP+) to K_v_1.1 wild-type (tdTomato+) progeny cells between the two genotypes at 1 mo old and 6 mo to 13 mo old. Two-way ANOVA followed by Sidak’s multiple comparisons test: genotype effect (*F*_1, 53_ = 7.0, *P* = 0.011), age effect (*F*_2, 53_ = 10, *P* = 0.0002), and genotype × age interaction (*F*_2, 53_ = 8.2, *P* = 0.0008); Sidak's multiple comparisons: 1 mo old (*P* = 0.96), 2 mo to 3 mo old (*P* < 0.0001), and 6 mo to 13 mo old (*P* = 0.93). Nestin-Cre;*Kcna1^+/−^*;MADM-6: 1 mo old (*n* = 12); 2 mo to 3 mo old (*n* = 11); 6 mo to 13 mo old (*n* = 9). Nestin-Cre;MADM-6 control: 1 mo old (*n* = 9); 2 mo to 3 mo old (*n* = 10); 6 mo to 13 mo old (*n* = 8). (*C*) Representative image with overlay and individual signals displaying K_v_1.1 null progenies (GFP+, green), K_v_1.1 wild-type progenies (tdTomato+, red), and K_v_1.1 heterozygous (GFP+ and tdTomato+, yellow) from the dentate gyrus of 8-mo-old Nestin-Cre;*Kcna1^+/−^*;MADM-6 mice. K_v_1.1 wild-type (1) and K_v_1.1 null (2) cells are marked (arrows). (Scale bar, 25 µm.) ****P < 0.0001. Data are presented as mean ± SEM. Data from 1-mo-old and 2- to 3-mo-old cohorts were originally published in figure 1 of Chou et al. (12).

In our previous studies, we performed MADM (33–35) with heterozygous K_v_1.1 (*Kcna1^+/−^*) mice, in which sparse somatic recombination driven by constitutively active Nestin-Cre generates a subpopulation of neural stem cells that lack K_v_1.1 (Nestin-Cre;*Kcna1^+/−^*;MADM-6) (Fig. 3*A*) (12). Homozygous K_v_1.1 null neural stem cell lineages are marked with GFP, and homozygous K_v_1.1 wild-type neural stem cell lineages are marked with tdTomato. Using this model, we observed ∼180% increase in progeny neurons from K_v_1.1 null neural stem cells in the dentate granule cell layer of 2- to 3-mo-old Nestin-Cre;*Kcna1^+/−^*;MADM-6 mice (*P* < 0.0001) [Fig. 3 *B* and *C*; data from 1-mo-old and 2- to 3-mo-old cohorts originally published in figure 1 of Chou et al. (12)]. This is consistent with our findings from K_v_1.1 null mice and K_v_1.1 cKO mice that loss of K_v_1.1 in neonatal neural stem cells promotes neonatal radial glia-like neural stem cell proliferation and neuronal production (*SI Appendix*, Fig. S3) (12). In 2- to 3-mo-old Nestin-Cre;*Kcna1^+/−^*;MADM-6 mice, presumably, a large population of neonatal-born neurons remains, as neonatal-born neurons begin apoptosis ∼2 mo after birth (57–59). However, once the mice have reached 6 mo to 13 mo of age, neonatal-born K_v_1.1 null progeny neurons would have been trimmed via apoptosis and therefore appear as a smaller portion of the GFP+ population. Indeed, we found no increase in K_v_1.1 null progeny neurons in the dentate granule cell layer of 6- to 13-mo-old mice (Fig. 3 *B* and *C*). As the loss of K_v_1.1 negatively impacts adult neurogenesis (Fig. 2), we conclude that adult-born K_v_1.1 null progeny neurons are unable to adequately replenish the GFP+ population. Thus, the transient increase of K_v_1.1 null progeny neurons in the MADM mice heterozygous for K_v_1.1 null mutation supports the hypothesis that K_v_1.1 plays an age-dependent role in adult neurogenesis.

### Mice with Conditional Knockout of K_v_1.1 from Adult Neural Stem Cells Display Impairments in Contextual Fear Conditioning.

**Fig. 4.**
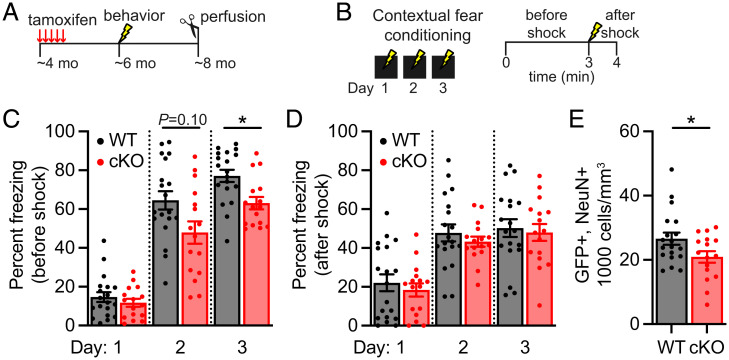
K_v_1.1 cKO mice display impairments in contextual fear conditioning. (*A*) Behavioral paradigm for assessing learning and memory in K_v_1.1 cKO mice and K_v_1.1 WT mice. Tamoxifen was injected for five consecutive days in ∼4-mo-old mice. Contextual fear conditioning started at ∼6.5 wk post tamoxifen injection. Mice were perfused at ∼8 mo of age. (*B*) Protocol for contextual fear conditioning. On days 1 to 3, the mice were shocked in the fear context. Percent freezing before the shock (0 min to 3 min) and after the shock (3 min to 4 min) were measured. (*C*) Quantification of percent freezing before shock on days 1 to 3, revealing a modest impairment of contextual fear learning and memory in K_v_1.1 cKO mice (*n* = 16) compared to K_v_1.1 WT mice (*n* = 19). Linear mixed model with REML and Geisser–Greenhouse correction followed by Sidak’s multiple comparisons test on days 1 to 3: genotype effect (*F*_1, 33_ = 7.5, *P* = 0.010), day effect (*F*_1.8, 61_ = 197, *P* < 0.0001), and genotype × day interaction (*F*_2, 66_ = 2.9, *P* = 0.060); Sidak’s multiple comparisons: day 1 (*P* = 0.75), day 2 (*P* = 0.098), and day 3 (*P* = 0.010). (*D*) Quantification of percent freezing after shock on days 1 to 3, revealing no difference between K_v_1.1 cKO mice and K_v_1.1 WT mice. Linear mixed model with REML and Geisser–Greenhouse correction followed by Sidak’s multiple comparisons test on days 1 to 3: genotype effect (*F*_1, 33_ = 0.54, *P* = 0.47), day effect (*F*_1.7, 55_ = 59, *P* < 0.0001), and genotype × day interaction (*F*_2, 66_ = 0.077, *P* = 0.93); Sidak’s multiple comparisons: day 1 (*P* = 0.88), day 2 (*P* = 0.77), and day 3 (*P* = 0.98). (*E*) Quantification (cells per cubic millimeter) of GFP+, NeuN+ cells to assess the amount of adult-born granule cells in the dentate gyrus of K_v_1.1 cKO and K_v_1.1 WT behavioral cohorts. Neurogenesis was decreased in K_v_1.1 cKO mice compared to K_v_1.1 WT mice (*P* = 0.037). Unpaired two-tailed Student’s *t* test with Welch’s correction. *P* < 0.10 indicated; *P < 0.05. Data are presented as mean ± SEM.

The hippocampus is important for contextual learning and memory; decreased hippocampal neurogenesis has been found to impair contextual fear conditioning, where mice learn to associate an environment with fear (60–63), and pattern separation, where they learn to discriminate between two similar contexts (40, 64, 65). Since the deletion of K_v_1.1 in adult neural stem cells reduced adult hippocampal neurogenesis (Fig. 2), we hypothesized that K_v_1.1 cKO mice would have learning and memory deficits compared to K_v_1.1 WT mice. We conducted behavioral tests on K_v_1.1 cKO mice starting at ∼4 mo of age when adult neurogenesis is less variable than at 2 mo of age (66). We injected ∼4-mo-old K_v_1.1 cKO mice with tamoxifen for five consecutive days to induce Cre expression and K_v_1.1 deletion in more neural stem cells (Fig. 4*A*). In control behavioral experiments, we did not find statistically significant differences between K_v_1.1 cKO and K_v_1.1 WT mice in the elevated plus maze, open field, and hotplate test (*SI Appendix*, Fig. S5). These results indicate that the mobility, anxiety, and pain perception of K_v_1.1 cKO mice are not different from those of K_v_1.1 WT mice. To test for the effects of K_v_1.1 cKO on hippocampus-dependent learning and memory, we used a protocol with three segments to first examine contextual fear conditioning, then contextual recall and generalization, and, finally, contextual discrimination (pattern separation) (64, 67).

First, mice underwent 3 d of contextual fear conditioning where they learned to associate the fear context with a foot shock. On days 1 to 3, they were placed in the fear context, a single foot shock was administered 3 min into the session, and mice were removed 1 min after the shock. Percent freezing before (0 min to 3 min) and after (3 min to 4 min) the foot shock was measured daily (Fig. 4*B*). The two genotypes did not display differences in percent freezing before the foot shock on day 1, showing that they have similar freezing levels at baseline (Fig. 4*C*). Interestingly, we found that K_v_1.1 cKO mice appeared to have reduced expression of contextual fear memory (genotype effect [*F*_1, 33_ = 7.5, *P* = 0.010], day effect [*F*_1.8, 61_ = 197, *P* < 0.0001], and genotype × day interaction [*F*_2, 66_ = 2.9, *P* = 0.060]), which manifested as a ∼25% and ∼20% reduction in freezing time prior to foot shock as compared to K_v_1.1 WT on day 2 and day 3 (post hoc analysis of linear mixed-model using Sidak’s multiple comparisons test: day 2 [*P* = 0.10], and day 3 [*P* = 0.010]) (Fig. 4*C*). Given that there was no difference between the two genotypes in percent freezing following the shock on days 1 to 3 (Fig. 4*D*), this study indicates that K_v_1.1 cKO mice exhibited a mild deficit in contextual fear conditioning.

Next, we demonstrated that both genotypes generalized their learned fear to a similar novel neutral context (*SI Appendix*, Fig. S6 *A*–*C*), thus allowing us to proceed to the third segment of our experiment and assess their ability to discriminate between the two contexts. In the pattern separation task on days 6 to 19, mice were placed in the two contexts daily, and shock was again administered in the fear context. Percent freezing was averaged for each 2-d block, to reduce variability (*SI Appendix*, Fig. S6*D*). As the mice learned to discriminate between the two contexts, they froze more in the fear context than the neutral context. We found no significant genotype effect in the pattern separation test (*SI Appendix*, Fig. S6 *E*–*I*).

To validate that the modest effect on contextual fear conditioning we observed was due to decreased adult hippocampal neurogenesis in K_v_1.1 cKO mice, we collected brain tissues from the behavioral cohorts and stained for NeuN to assess the extent of neurogenesis in the GFP+ lineages with tamoxifen-induced Cre expression and, in K_v_1.1 cKO cohort, deletion. We found that adult neurogenesis was decreased by ∼20% in K_v_1.1 cKO mice as compared to K_v_1.1 WT controls (*P* = 0.037) (Fig. 4*E*). These observations are consistent with previous studies reporting diminished adult neurogenesis resulting in deficient contextual fear conditioning (60–63). Whereas past studies using X-ray irradiation to reduce adult neurogenesis by more than ∼90% have produced deficits in pattern separation (40, 64), the ∼20% decrease in adult neurogenesis caused by the loss of K_v_1.1 is probably insufficient to impair their performance in the pattern separation test. In summary, these results indicate that deletion of K_v_1.1 hinders adult neurogenesis, resulting in mild impairments in contextual fear conditioning.

## Discussion

Adult hippocampal neurogenesis is critical for learning and memory, and altered adult neurogenesis has been implicated in aging and neurological disorders (1–6). Although voltage-gated ion channels have been shown to modulate the membrane potential and cell dynamics of neural stem and progenitor cells during vertebrate and invertebrate neurodevelopment (9–11, 68–70), the role of bioelectric signaling in adult hippocampal neurogenesis has only recently begun to be explored. In our previous study, we found that genetic ablation of K_v_1.1 depolarizes neonatal neural progenitor cells and increases their proliferation through enhanced TrkB signaling in neonatal hippocampal development (12). As K_v_1.1 null mice develop seizures that could impact adult neurogenesis (27, 29–31, 36, 37) and confound the possible impact of K_v_1.1 deletion on adult neurogenesis, we developed a strategy for inducible conditional knockout of K_v_1.1 from neural stem cells of adult mice (*SI Appendix*, Fig. S1*A*). These K_v_1.1 cKO mice allowed us to examine the role of K_v_1.1 at different stages of adult neurogenesis and better elucidate the role of K_v_1.1 during neonatal and adult development.

**Fig. 5.**
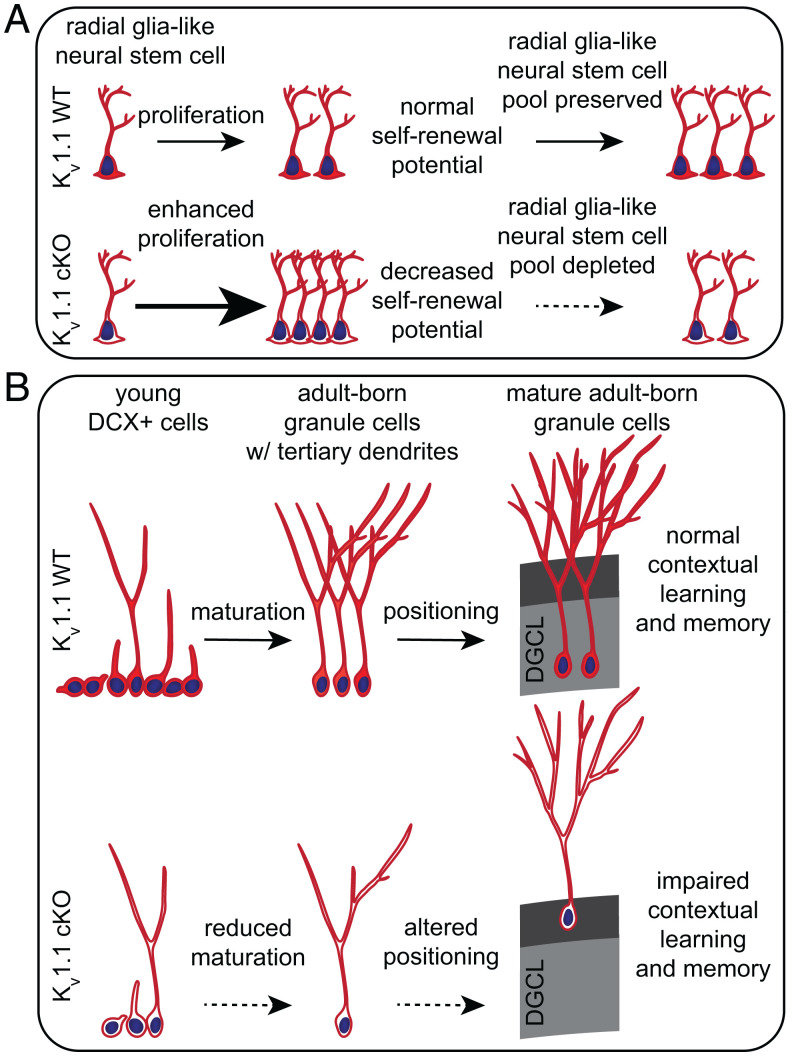
Model of impaired adult hippocampal neurogenesis in K_v_1.1 cKO mice with tamoxifen-induced Cre expression and K_v_1.1 removal from neural stem cells. (*A*) During early stages of adult hippocampal neurogenesis, removal of K_v_1.1 from neural stem cells enhances radial glia-like neural stem cell proliferation, leading to an initial increase of radial glia-like neural stem cells at 4 wk post tamoxifen injection (*Center*). Overproliferation diminishes the radial glia-like neural stem cells’ self-renewal potential and depletes radial glia-like neural stem cells in K_v_1.1 cKO mice, resulting in decreased radial glia-like neural stem cells by 8 wk post tamoxifen injection (*Right*). (*B*) In later stages of neurogenesis, K_v_1.1 cKO mice have fewer DCX+ cells (*Left*), and these DCX+ cells have deficits in development with a reduction in the percentage and amount of adult-born granule cells with tertiary dendrites (*Center*). Fewer mature adult-born granule cells are produced in K_v_1.1 cKO mice, and these adult-born granule cells are more likely to be mispositioned in the outer third of the DGCL (*Right*). Thus, K_v_1.1 plays an important role in proper adult-born granule cell maturation and integration into the DGCL and in preserving hippocampus-dependent contextual learning and memory.

During early steps of adult hippocampal neurogenesis, K_v_1.1 is important for preserving the radial glia-like neural stem cell (type 1 cell) pool (Fig. 5*A*), which is maintained by a delicate balance between radial glia-like neural stem cell quiescence and activation (71). Initially, radial glia-like neural stem cells without K_v_1.1 rapidly divide to 1) self-renew, generating more quiescent radial glia-like neural stem cells (Fig. 1*C*), and 2) differentiate, likely producing more type 2a cells (*SI Appendix*, Fig. S4*B*). However, radial glia-like neural stem cells have varying self-renewal capacity. Over time, increased proliferation in the absence of K_v_1.1 may become unsustainable and might exhaust the radial glia-like neural stem cells with limited proliferative potential as they undergo terminal differentiation and are eliminated from the progenitor pool (72, 73). Interestingly, the switch between radial glia-like neural stem cell quiescence and activation can be regulated by network activity of glutamatergic mossy cells and long-range GABAergic neurons. Ablation of both cell types can produce a similar initial activation followed by depletion of radial glia-like neural stem cells (13, 15, 18). In addition to regulation via synaptic inputs, our study suggests that radial glia-like neural stem cells may rely on K_v_1.1 channel activity to prevent their subsequent activation and depletion (*SI Appendix*, Fig. S2 and Fig. 1). Perhaps loss of K_v_1.1 depolarizes adult neural stem and progenitor cells and promotes proliferation through increased TrkB signaling, as we previously observed in neonatal neurogenesis (12).

Our study further reveals that loss of K_v_1.1 in the neural stem cell lineage impedes adult-born granule cell development in later stages of adult neurogenesis. In K_v_1.1 cKO mice, there was a reduction of DCX-expressing cells as well as impairment of adult-born granule cell maturation and positioning (Fig. 2 *A–F*). Notably, aberrant positioning of adult-born granule cells has been observed in mouse models of traumatic brain injury, schizophrenia, and neurodegeneration (54–56). Failing to properly mature and position themselves, young adult-born granule cells produced from neural stem cells lacking K_v_1.1 likely struggle to integrate into the dentate gyrus circuit, resulting in decreased survival of new adult-born granule cells and reduced mature adult-born granule cells (NeuN+) in K_v_1.1 cKO mice (Fig. 2 *G* and *H*). Interestingly, a recent study discovered that postmortem samples from patients with neurodegenerative disease display a similar increase in radial glia-like neural stem cells and impairment in adult-born granule cell maturation and positioning (74). In mouse models, failures in adult neurogenesis often lead to deficits in hippocampus-dependent learning and memory (38, 40, 60–65). Indeed, K_v_1.1 cKO mice have moderately diminished contextual fear learning and memory (Fig. 4). Together, these results underscore the critical function of K_v_1.1 in maintaining adult-born granule cell maturation and positioning for proper integration into the dentate gyrus circuit and preservation of hippocampus-dependent learning and memory (Fig. 5*B*).

Our understanding of the role of K_v_1.1 in adult neurogenesis also helps to clarify the role of K_v_1.1 in neonatal neurogenesis. Given that we observed an initial increase in radial glia-like neural stem cell proliferation when K_v_1.1 was removed from adult neural stem cells, the increase in neonatal neurogenesis observed when K_v_1.1 is removed from neonatal neural stem cells in both K_v_1.1 null mice (12) and K_v_1.1 cKO mice (*SI Appendix*, Fig. S3) is likely to have arisen from neonatal radial glia-like neural stem cell overproliferation. Unlike adult neural stem and progenitor cells, neonatal neural stem and progenitor cells have extensive proliferative potential (43, 44) and produce neurons with delayed cell death (57–59). These properties of neonatal neurogenesis allow the neuronal progenies to last for a longer period, which would account for the initial increase in K_v_1.1 null progeny of 2- to 3-mo-old Nestin-Cre;*Kcna1^+/−^*;MADM-6 mice (Fig. 3) before neuron death starting at 2 mo postmitosis (57–59). As the MADM mice age, neonatal-born K_v_1.1 null neurons begin cell death, and radial glia-like neural stem cells lacking K_v_1.1 become depleted, as in the case of adult K_v_1.1 cKO mice. Together, these factors contribute to the transient increase of K_v_1.1 null progeny neurons in 2- to 3-mo-old but not 6- to 13-mo-old Nestin-Cre;*Kcna1^+/−^*;MADM-6 mice (Fig. 3). These results support our model that K_v_1.1 functions to maintain hippocampal neurogenesis at multiple developmental timepoints.

In summary, we demonstrate that K_v_1.1 is important for adult hippocampal neurogenesis and hippocampus-dependent contextual learning and memory. K_v_1.1 likely regulates the neurogenic niche by preventing the overproliferation and depletion of radial glia-like neural stem cells. As young adult-born granule cells develop, loss of K_v_1.1 impedes their dendritic maturation and positioning, likely hampering their integration into the circuit. These developmental failures in K_v_1.1 cKO mice contribute to decreased adult-born granule cells and mild deficits in contextual fear learning and memory (Fig. 5). Our findings provide the basis for future studies to elucidate the impact of K_v_1.1 regulation on adult neurogenesis under normal and pathophysiological conditions.

## Materials and Methods

### Animals.

All experiments were approved by the Institutional Animal Care and Use Committees of the University of California, San Francisco and Academia Sinica. Two to five mice per cage were maintained in a temperature-controlled environment on a 12-h light/dark cycle with ad libitum access to food and water.

*Kcna1^fl/fl^* mice (41) were obtained from E.G.’s laboratory at Southern Methodist University, Dallas, TX. Nestin-Cre^ERT2^ mice (38–40) were obtained from Mazen Kheirbek’s laboratory at University of California, San Francisco, CA. PC::G5-tdT mice (42) were obtained from the Jackson Laboratory. We utilized the PC::G5-tdT reporter line because its Cre reporter alleles are located on a different chromosome (Chr 11) from *Kcna1* (Chr 6). These three lines were bred together to create K_v_1.1 cKO mice and maintained on a C57BL/6 background.

To create the Nestin-Cre;*Kcna1^+/−^*;MADM-6 mice, the lines bred together were *Kcna1^−/−^* mice (31), obtained from Bruce Tempel’s laboratory at the University of Washington, Seattle, WA; Nestin-Cre (Tg(Nes-cre)1Kln) (75), obtained from the Jackson Laboratory; and MADM-6 mice with Rosa26^GT^ (Gt(ROSA)26Sor^tm6(ACTB-EGFP*,-tdTomato)^) and Rosa26^TG^ (Gt(ROSA)26Sor^tm7(ACTB-EGFP*)^) (76), obtained from Liqun Luo's laboratory at Stanford University, Palo Alto, CA. All these mice were maintained on an ICR background. MADM experiments were performed as previously described (12).

### Drug Administration.

For neonatal tamoxifen (MilliporeSigma) administration, tamoxifen was dissolved in 100% corn oil (MilliporeSigma) at 10 mg of tamoxifen per mL. At P0, pups were injected once subcutaneously with 30 μL of 10 mg of tamoxifen per mL. For adult tamoxifen administration, tamoxifen was dissolved in a solution of 10% ethanol (200 proof, VWR) in corn oil at 20 mg of tamoxifen per mL. Adult 8-wk-old mice were intraperitoneally injected with 100 mg of tamoxifen per kg of body weight once per day for three consecutive days for single-cell suspension and immunohistochemistry. Adult ∼4-mo-old mice were injected with 100 mg of tamoxifen per kg of body weight once per day for five consecutive days for behavioral experiments. BrdU (MilliporeSigma) was dissolved in sterile normal (0.9%) saline at 5 mg of BrdU per mL. One dose of 50 mg of BrdU per kg of body weight was injected subcutaneously at P7.

### Single-Cell Suspension for qPCR and Electrophysiology.

At 2 wk post tamoxifen injection, mice were euthanized, and their brains were transferred into ice-cold 1× Hanks’ balanced salt solution (HBSS). Under a dissecting microscope, dentate gyri were isolated and pooled from four mice of the same genotype. The Neural Tissue Dissociation (P) Kit (Miltenyi Biotec) was used to dissociate tissue into single cells. After a final HBSS wash, the cell pellet was resuspended in 800 μL of Hibernate A Low Fluorescence medium (BrainBits). Detailed methods for qPCR and electrophysiology are included in *SI Appendix*, *SI Materials and Methods*.

### Immunostaining.

Mice were anesthetized with isoflurane (Henry Schein Animal Health) before transcardial perfusion with cold phosphate-buffered saline (PBS) followed by cold 4% paraformaldehyde (PFA) in PBS. Brains were removed, postfixed overnight in 4% PFA for ∼24 h at 4 °C, washed in PBS, and immersed in 30% sucrose in PBS for a minimum of 48 h at 4 °C for cryoprotection. Then, the brains were frozen in optimal cutting temperature compound (Fisher Scientific). Free-floating 30-µm sagittal sections were collected from the lineage tracing cohort and coronal sections were collected from the behavior cohort into PBS using a cryostat (Lecia CM3050 S, Leica Microsystems). Afterward, the sections were transferred into cryoprotectant (30% ethylene glycol, 30% glycerol, 40% PBS) and stored at −20 °C.

For immunohistochemistry, sections were removed from the cryoprotectant, washed 3 × 10 min in PBS, treated with 15 min of 0.5% triton in PBS, and transferred into blocking buffer (5% normal donkey serum, 1% bovine serum albumin, 0.05% triton in PBS) for 1 h at room temperature. Then, they were incubated in primary antibodies overnight at 4 °C. The next day, they were washed 3 × 10 min with 0.05% triton in PBS and incubated in secondary antibodies for 1 h at room temperature. After 3 × 10 min PBS washes, they were mounted using Fluoromount G mounting media with DAPI (Southern Biotech) on Superfrost Plus microscope slides (Fisher Scientific). Detailed methods for tissue processing and treatments are included in *SI Appendix*, *SI Materials and Methods*.

Primary antibodies were diluted in blocking buffer as listed: chicken GFP antibody (GFP-1020, Aves Labs) at 1:1,000, mouse MCM2 antibody (610701, BD Biosciences) at 1:500, rabbit Sox2 antibody (ab97959, Abcam) at 1:500, goat GFAP antibody (ab53554, Abcam) at 1:1,000, rabbit NeuN antibody (12943, Cell Signaling Technology) at 1:1,000, guinea pig DCX antibody (AB2253, MilliporeSigma) at 1:1,000, and mouse BrdU antibody (B35128, ThermoFisher) at 1:500.

Secondary antibodies were diluted in blocking buffer at 1:1,000 as listed: donkey anti-mouse Alexa Fluor 488 (A21202, ThermoFisher Scientific), donkey anti-chicken Cy3 (703-165-155, Jackson ImmunoResearch Laboratories, Inc.), donkey anti-rabbit Alexa Fluor-594 (711-585-152, Jackson ImmunoResearch Laboratories, Inc.), donkey anti-rabbit Alexa Fluor-647 (A31573, ThermoFisher Scientific), donkey anti-goat Alexa Fluor Plus 647 (A32849, ThermoFisher Scientific), and donkey anti-guinea pig Alex Flour-647 (706-605-148, Jackson ImmunoResearch Laboratories, Inc.).

### Microscopy and Sampling.

One of every 10 sections in a series spanning the entire dentate gyrus was imaged on a confocal microscope (Leica Sp8) using a 40× or 63× Harmonic Compound Plan Apochromat oil Confocal Scanning 2 objective. For lineage analysis, a z-stack of five 3-µm steps was collected per dentate gyrus section for one hemisphere. The dorsal dentate gyrus was quantified, given that Cre expression was variable in the ventral dentate gyrus (77). The upper third of the dentate granule cell layer was defined as being within two dentate granule cell layers of the molecular layer. For behavioral analysis, two sections from both hemispheres were quantified. Images were processed and analyzed using Fiji (ImageJ, NIH). Experimenters were blinded to the genotype of the mice during analysis.

### Behavioral Tests.

We did not observe overt differences in health between the two genotypes. Mice used for these experiments were healthy, without injuries that would interfere with behavioral testing. Behavioral data were obtained with the help of the Gladstone Behavioral Core. The experimenters were blinded to the genotype of the mice for all behavioral testing.

For behavior experiments, a cohort of adult ∼4-mo-old (age range 13 wk to 17 wk) mice were injected with tamoxifen once per day for five consecutive days. Five weeks after the last tamoxifen injection, behavioral testing began with the elevated plus maze, followed by the open field. Contextual conditioning and discrimination testing began at ∼6.5 wk post tamoxifen injection. Afterward, at ∼10.5 wk post tamoxifen injection, the hot plate test was conducted. Finally, mice were perfused at ∼17.5 wk post tamoxifen injection. Detailed methods for elevated plus maze, open field, contextual fear conditioning and discrimination, and hot plate behavioral tests are included in *SI Appendix*, *SI Materials and Methods*.

### Statistical Analyses.

All data were summarized as mean ± SEM. Comparisons between two genotypes were analyzed by unpaired two-tailed Student’s *t* tests. If the data did not meet the Student’s *t* test’s assumptions of normality and variance, the data were analyzed using the unpaired two-tailed Student's *t* test with Welch’s correction or the nonparametric Mann–Whitney *U* test, as indicated in the figure legends. Multiple group comparisons were assessed using one-way ANOVA with Holm–Sidak correction for multiple comparisons, two-way ANOVA followed by Sidak’s multiple comparisons test, or linear mixed-model with restricted maximum likelihood (REML) and Geisser–Greenhouse correction followed by Sidak’s multiple comparisons test, as indicated in the figure legends. The null hypothesis was rejected at *P* > 0.05. Data were analyzed using Prism 9 (GraphPad).

## Supplementary Material

Supplementary File

## Data Availability

All study data are included in the article and/or *SI Appendix*.
