# Supplementary material for: Kv1.1 preserves the neural stem cell pool and facilitates neuron maturation during adult hippocampal neurogenesis
Source: Proc Natl Acad Sci U S A. 2022 May 25;119(22):e2118240119. doi: 10.1073/pnas.2118240119 (PMC9295736; doi:10.1073/pnas.2118240119)
Supplement: Supplementary File [file pnas.2118240119.sapp.pdf]

PNAS
www.pnas.org

Supplementary Information for

K_v1.1 preserves the neural stem cell pool and facilitates neuron maturation during adult hippocampal neurogenesis

Author list:
Yuan-Hung Lin King[abcd], Chao Chen[bcd], John V. Lin King[ab], Jeffrey Simms[e], Edward Glasscock[f], Shi-Bing Yang[gh], Yuh-Nung Jan[bcd], Lily Y. Jan[bcd1]

[a]Neuroscience Graduate Program, University of California, San Francisco, CA 94143;
[b]Department of Physiology, University of California, San Francisco, CA 94143;
[c]Department of Biochemistry and Biophysics, University of California, San Francisco, CA 94143;
[d]Howard Hughes Medical Institute, University of California, San Francisco, CA 94143;
[e]Behavioral Core, Gladstone Institute of Neurological Disease, Gladstone Institutes, San Francisco, CA 94158;
[f]Department of Biological Sciences, Southern Methodist University, Dallas, TX 75275;
[g]Institute of Biomedical Sciences, Academia Sinica, Taipei, 115, Taiwan;
[h]Neuroscience Program of Academia Sinica, Academia Sinica, Taipei, 115, Taiwan;

[1]Lily Y. Jan
Email: lily.jan@ucsf.edu

This PDF file includes:

Supplementary text
Figures S1 to S6
SI References

Other supplementary materials for this manuscript include the following:

Not applicable

Supplementary Information Text

SI Materials and Methods

Cell sorting and qPCR

The single-cell suspension was incubated with LIVE/DEAD™ stain 633 nm (1:2000, Invitrogen) for 30 min at 4 ºC to identify dead cells. Cells were then filtered using a 40 µm strainer and sorted on the FACS Aria™ III Cell Sorter (BD Bioscience). Based on forward and side scatter, cells were gated to exclude debris and doublets. The cell population was selected based on the intensity of tdTomato fluorescence and LIVE/DEAD™ stain 633 nm.

Pooled tdTomato+ live cell mRNA was extracted using RNAqueous-Micro Total RNA Isolation Kit (Invitrogen). The qPCR reactions were run using PowerUp™ SYBR™ Green master mix (Applied Biosystems) along with 100 nM primers (IDT). Relative mRNA levels were determined using the $2^{-\Delta\Delta C_T}$ method (1). *Kcna1* mRNA levels were normalized by the mRNA levels of the house keeping *Gapdh* gene. The primer sequences used to amplify the target genes were: *Gapdh* forward (5'-TCACCACCATGGAGAAGGC-3'); *Gapdh* reverse (5'-GCTAAGCAGTTGGTGGTGCA-3'); *Kcna1* forward (5'-AGATCGTGGGCTCCTTGTGT-3'); *Kcna1* reverse (5'-ACGGGCAGGGCAATTGT-3').

Electrophysiology

Dissociated cells were plated onto coverslips coated with 0.01% poly-L-Lysine (MW 70,000-150,000, MilliporeSigma) and mouse laminin (10 µg per mL). After the cells were allowed to adhere for at least 30 min at room temperature, the coverslips were transferred into an artificial cerebrospinal fluid recording buffer that contained (in mM) 127 NaCl, 1.8 KCl, 10 HEPES, 1.3 $MgCl_2$, 2.4 $CaCl_2$, 15 Glucose, at pH 7.4 with HCl and 300-310 mOsm per kg. To record from $K_V1.1$ WT and $K_V1.1$ cKO cells, capillary glass pipettes were created from filamented borosilicate glass (O.D. × I.D., 1.10 × 0.86 mm, Sutter Instruments) and fire-polished to 6-10 mΩ resistance. These pipettes were backfilled with solution containing (in mM) 120 K-Gluconate, 15 KCl, 1.4 $MgCl_2$, 0.1 EDTA, 10 HEPES, 4 Mg-ATP, 0.3 Na_3-GTP, 7 Phosphocreatine, at pH 7.4 with KOH and 290-300 mOsm per kg. As indicated, this solution was supplemented with 10 nM Dendrotoxin K (Alomone Labs), which is a selective inhibitor of $K_V1.1$ at this concentration (2). Cells that had undergone Cre recombinase (Cre)-mediated recombination expressed tdTomato and were identified by red epifluorescence. Cell-attached patch-clamp electrophysiological recordings were then carried out at room temperature under laminar flow of artificial cerebrospinal fluid using a pressure-driven micro-perfusion system (SmartSquirt, Automate Scientific). An Axopatch 200B amplifier (Axon Instruments) was employed to collect the data, which was digitized via a Digidata 1550B (Axon Instruments). Voltage stimulus protocols were applied, and their evoked currents were measured on-line with pClamp10 (Molecular Devices), sampling data at 20 kHz and filtering it at 2 kHz. To measure cells' resting membrane potential, K^+ currents were evoked by applying a 50 ms voltage ramp from −100 mV to 100 mV. Current-voltage (I-V) relationships were analyzed off-line in pClamp and Prism (GraphPad). Because we set the pipette $[K^+]$ approximately equal to the intracellular $[K^+]$, the equilibrium potential for K^+ across the patch was approximately zero. Therefore, K^+ currents reversed direction when the pipette potential was equal to the membrane potential, allowing us to measure resting membrane potential (3, 4).

Immunostaining

If additional treatments were required, they were performed before the blocking step described in *Materials and Methods*. For MCM2 staining, which required antigen retrieval, sections were placed in sodium citrate buffer (10 mM sodium citrate, 0.05% tween 20, pH 6.0) for 3 h at 70 ºC in a convection drying oven (Yamato), then allowed to cool for 15 min on ice. For Bromodeoxyuridine (BrdU) visualization, DNA denaturation with 2N HCl was required. Sections were placed in 2N HCl for 30 min at 37 ºC. After 3 × 10 min PBS washes, sections were moved into blocking buffer.

Behavioral tests

Elevated plus maze

The elevated plus maze (Hamilton-Kinder, Poway, CA) consisted of two open arms (without walls, 15” long × 2” wide), two closed arms (with walls 6.5” tall), and an intersection of the arms (2” × 2” wide). Mice were habituated in the testing room under dim light for 1 h before being placed into the intersection of the two arms in the elevated plus maze and allowed to explore freely for 10 min. Total distance traveled and time on the open and closed arms were recorded by the system using infrared photobeam breaks. The maze was cleaned with 70% ethanol between animals.

Open field

Mice were habituated in the testing room under normal light for 1 h before being placed into the center of the arena and allowed to explore freely for 15 min. The Flex-Field/Open Field Photobeam Activity System (San Diego Instruments, San Diego, CA) was used for this experiment. It consisted of a clear acrylic chamber (41 cm × 41 cm × 30 cm) inside sound and light attenuating shells to eliminate external stimuli. Within the chamber, there were two 16 × 16 photobeam arrays that automatically detect horizontal and vertical movements. Total movements, ambulatory movement (disruption of three or more consecutive photobeams), fine movement (repeated disruption of the same two photobeams), center/total movement, and rearing were collected by the system for analysis. The arena was cleaned with 70% ethanol between animals.

Contextual fear conditioning and discrimination testing

The pattern separation task was adapted from (5) and (6). A conditioning chamber (Med Associates Inc) was used for the experiment. The fear context consisted of background noise from a 60 dB fan and scent of 70% Windex sprayed into the bedding pan. The neutral context consisted of a black A-frame insert, no background noise, and 10% Simple Green scent.

For contextual conditioning experiments, mice were placed in the fear context for a 4-min session for three consecutive days. There were no stimuli for the first 3 min baseline period followed by a single mild foot shock (0.45 mA) lasting 2 s. Animals were exposed to one shock per session per day followed by a 1-min interval before being returned to their home cage. Conditioning was assessed by measuring "freezing" behavior. “Freezing” was defined as a defensive posture characterized by lack of all movement except that required for respiration.

Contextual memory recall and generalization tests were conducted over the next 2 d (days 4 and 5) in the absence of any foot shocks. On day 4, the mice were placed in the fear training context in the morning for a 4-min session. In the afternoon, animals were introduced to the neutral no-shock context for a 4-min session to provide a measure of context generalization. On day 5 of testing, the mice were placed in the neutral context in the morning and the fear context in the afternoon. A 4-h delay separated the two sessions in which the mice were returned to the colony room. The percent freezing behavior exhibited in the first 3 min of each 4-min session was quantified.

For measurements of pattern separation between the two contexts, mice were again given two 4-min sessions each day over the course of the next fourteen consecutive days except that during this phase of the testing, a single foot shock was again delivered to the mice in the fear context after an initial 3-min baseline period. No foot shocks were delivered while the mice were in the neutral context. Discrimination learning between the two contexts was determined by comparing the percent freezing behavior exhibited in the first 3 min of each 4-min testing session in each context. Percent freezing was averaged from 2 d to form a block. The discrimination ratio is (percent freezing in fear context − percent freezing in neutral context) / (percent freezing in fear context + percent freezing in neutral context).

Hot plate

Mice were habituated in the testing room under normal light for 1 h before being placed on top of the hot plate in a clear, open-ended cylindrical enclosure. The temperature was set to 52 ºC. The latency to respond (for example a hindpaw lick, hindpaw flick, or jump) was measured. The mouse

was immediately removed from the hot plate after responding. The maximum latency to respond was 30 s to prevent injury.

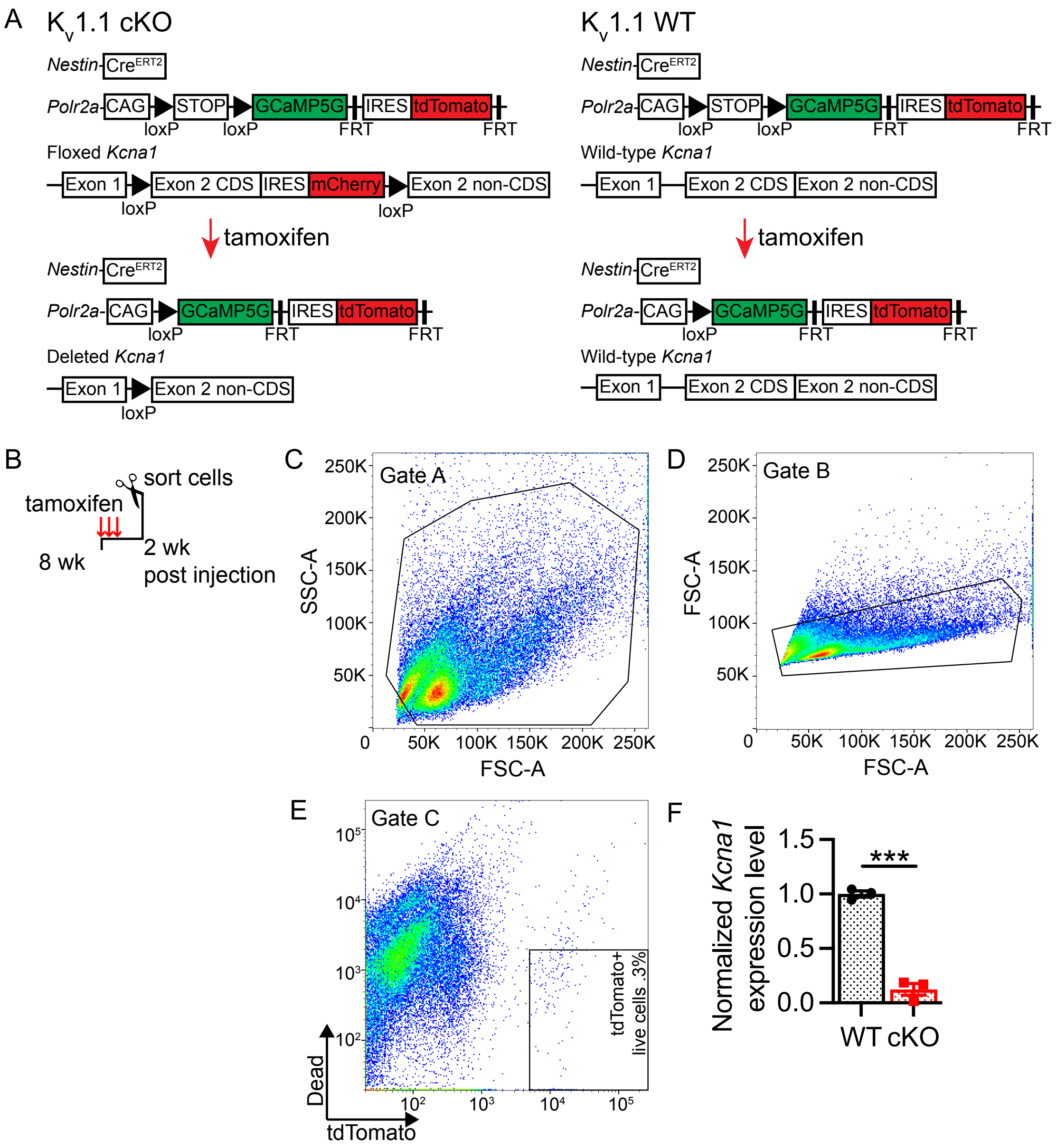

Fig. S1. Generation of Kv1.1 conditional knockout (cKO) mice and validation of *Kcna1* gene deletion by qPCR. (*A*) Strategy for cell type-specific Kv1.1 knockout with temporal control via tamoxifen administration. We bred Nestin-Cre^{ERT2} mice, which expressed a tamoxifen-inducible Cre in neural stem cells, with *Kcna1*$^{fl/fl}$ mice and PC::G5-tdT reporter mice to create Kv1.1 cKO mice. Within the *Kcna1*$^{fl/fl}$ allele, the *Kcna1* exon 2 coding sequence (CDS) is flanked by loxP sites and removed by tamoxifen-activated Cre recombinase. However, mCherry fluorescence in *Kcna1*$^{fl/fl}$ mice was not visible even with antibody amplification. Because Cre recombinase also allows for both tdTomato and GCaMP5G expression in the PC::G5-tdT transgene, we quantified the expression of these markers as a proxy for Cre expression. To establish a Kv1.1 wild-type (WT) control line, we bred Nestin-Cre^{ERT2} mice and PC::G5-tdT mice with *Kcna1* wild-type mice, thereby controlling for tamoxifen exposure as well as Cre recombinase and reporter expression. (*B*) Protocol for validation of Kv1.1 cKO by qPCR. We injected 8-wk-old mice with tamoxifen for three consecutive days to induce Cre expression and *Kcna1* deletion. After 2 wk, we dissected out the dentate gyrus, suspended the cells, and sorted out tdTomato+ cells for qPCR to determine if *Kcna1* has been deleted. (*C–E*) Representative image demonstrating fluorescence-activated cell sorting strategy for

isolation of tdTomato+ lineage. (*C*) Cells were first gated based on forward scatter area (FSC-A) and side scatter area (SSC-A) properties to eliminate debris (Gate A). (*D*) Second, cell doublets were excluded based on the area and width of the forward scatter area (Gate B). (*E*) Third, the cell population was selected based on the intensity of the tdTomato fluorescence and LIVE/DEAD™ stain 633 nm (Gate C). In total, 0.3% of the sorted cells were collected. (*F*) QPCR analysis of sorted tdTomato+ live cells. Cells were pooled, and qPCR analysis showed that *Kcna1* mRNA expression was decreased in the $K_v1.1$ cKO mice (n = 3) compared to $K_v1.1$ WT mice (n = 3) (P = 0.0001). Unpaired two-tailed Student's t test. *** $P < 0.001$. Data are presented as mean $\pm$ SEM.

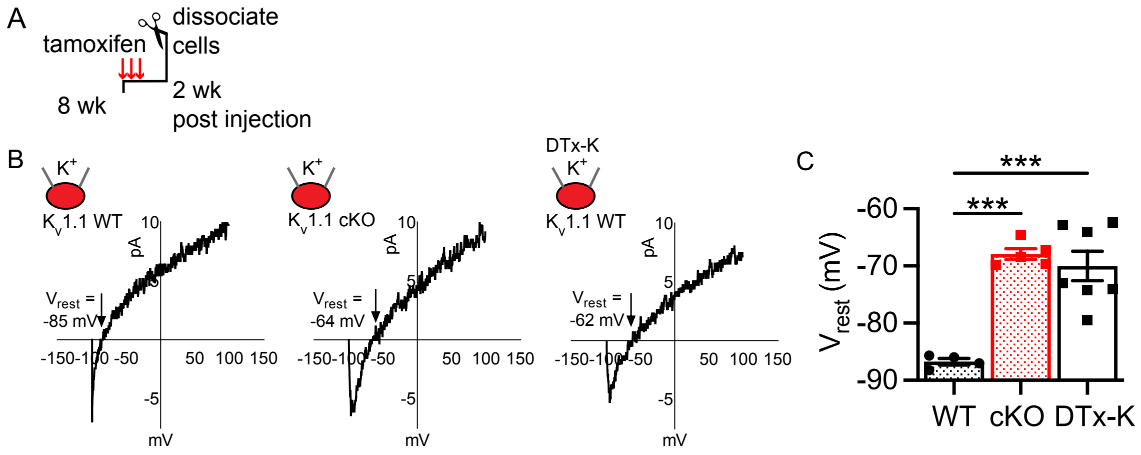

Fig. S2. Loss of Kᵥ1.1 function in Kᵥ1.1 cKO cells. (*A*) Protocol for validation of Kᵥ1.1 cKO by cell-attached patch recording. We injected 8-wk-old mice with tamoxifen for three consecutive days to induce Cre expression and *Kcna1* deletion. After 2 wk, we dissected out the dentate gyrus, suspended and plated the cells, and carried out cell-attached patch recordings from tdTomato+ cells. (*B*) Representative current-voltage (I-V) curves of Kᵥ1.1 WT cell (*Left*), Kᵥ1.1 cKO cell (*Center*), and Kᵥ1.1 WT cells treated with Dendrotoxin-K (DTx-K, 10 nM) (*Right*). Resting membrane potentials for each cell type are indicated. (*C*) Summary of resting membrane measurements. TdTomato+ Kᵥ1.1 cKO cells ($n = 5$) and Kᵥ1.1 WT cells treated with DTx-K ($n = 7$) displayed depolarized resting membrane potential compared with Kᵥ1.1 WT cells ($n = 4$). One-way ANOVA with Holm-Sidak correction for multiple comparisons: ($F_{2,13} = 21$, $P < 0.0001$); multiple comparisons: WT vs. cKO ($P = 0.0002$), WT vs. DTx-K ($P = 0.0002$), and cKO vs. DTx-K ($P = 0.47$). ***$P < 0.001$. Data are presented as mean ± SEM.

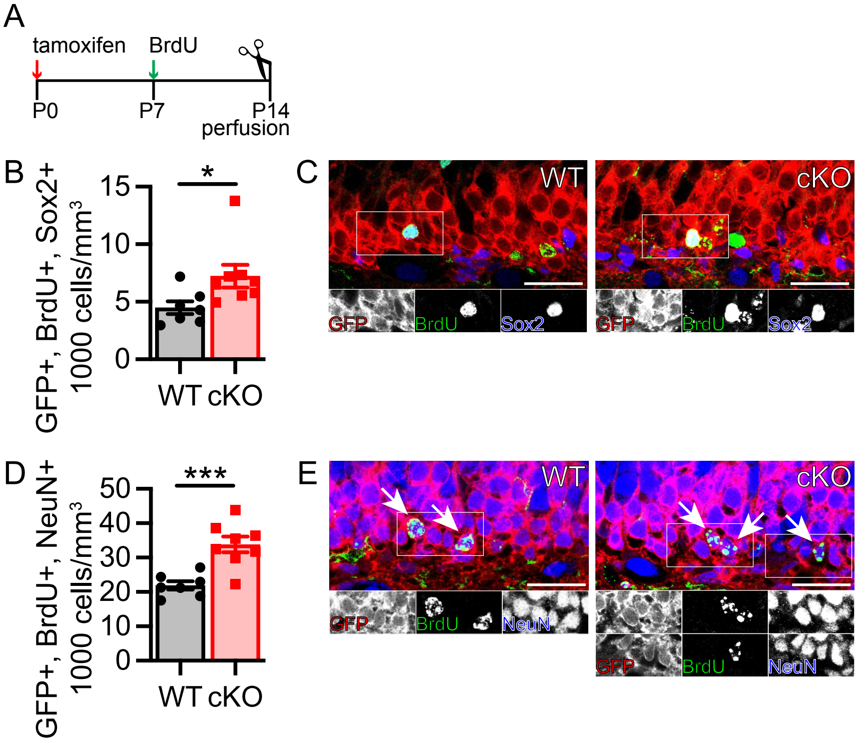

Fig. S3. Neonatal deletion of Kv1.1 increases hippocampal neurogenesis**.** (*A*) Neonatal lineage tracing protocol. Tamoxifen was injected at postnatal day 0 (P0) to delete Kv1.1 in neural stem cells, and BrdU was injected at postnatal day 7 (P7) for lineage tracing. Brains were harvested at postnatal day 14 (P14). (*B*) Quantification (cells per cubic millimeter) at P14 of GFP+ neural stem and progenitor cells (GFP+, BrdU+, Sox2+) produced from P7 dividing cells. An increase of GFP+, BrdU+, and Sox2+ cells was observed in Kv1.1 cKO mice (n = 8) compared to Kv1.1 WT mice (n = 7) (P = 0.036). (*C*) Representative image showing expression of GFP (red), BrdU (green), and Sox2 (blue) in the Kv1.1 WT (*Left*) and Kv1.1 cKO (*Right*) dentate gyrus. GFP, BrdU, and Sox2 staining within the boxed area are individually shown (*Below*). (Scale bar, 25 μm.) (*D*) Quantification (cells per cubic millimeter) at P14 of neurons (GFP+, BrdU+, NeuN+) produced from P7 dividing cells. Kv1.1 cKO mice displayed more neurogenesis compared to Kv1.1 WT mice (P = 0.0007). (*E*) Representative image displaying expression of GFP (red), BrdU (green), and NeuN (blue) in the Kv1.1 WT (*Left*) and Kv1.1 cKO (*Right*) dentate gyrus. GFP, BrdU, and NeuN signals are individually shown (*Below*). GFP+, BrdU+, and NeuN+ cells are marked (arrows). (Scale bar, 25 μm.) (*B* and *D*) Unpaired two-tailed Student's t test. * $P < 0.05$, *** $P < 0.001$. Data are presented as mean ± SEM.

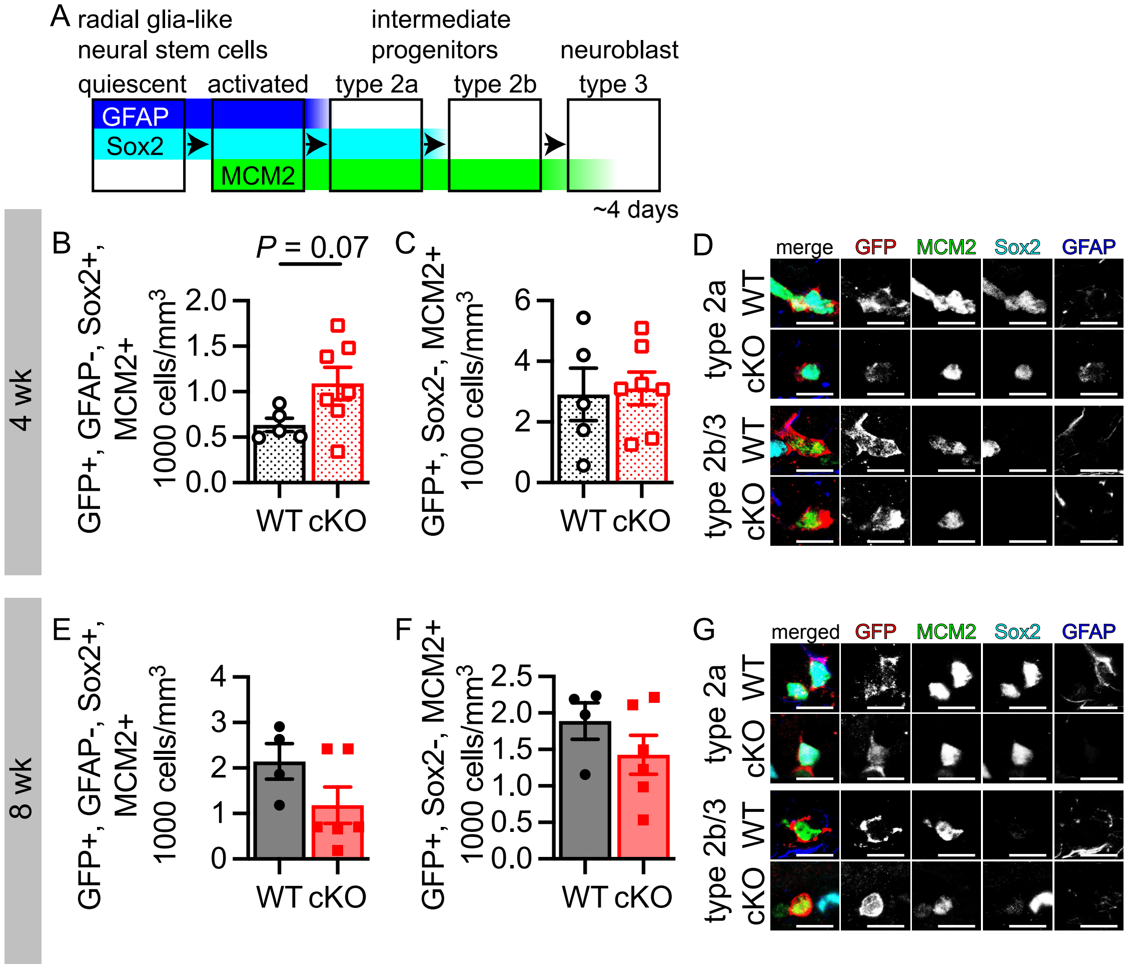

Fig. S4. Deletion of $K_v1.1$ in adult neural stem cells results in a trend toward increased glia-like intermediate neural progenitor cells (type 2a cells) at 4 wk post tamoxifen injection. (*A*) Cell marker expression during adult hippocampal neurogenesis. Quiescent radial glia-like neural stem cells are GFAP+ and Sox2+ and start expressing MCM2 when they become activated. As radial glia-like neural stem cells develop into type 2a cells they lose their GFAP expression but remain Sox2+ and MCM2+. Sox2 expression is lost as type 2a cells become more neuron-like intermediate neural progenitors (type 2b cells). They continue to express MCM2 until they become postmitotic neuroblasts (type 3 cells). (*B* and *C*) Quantification (cells per cubic millimeter) of type2a cells (GFP+, GFAP−, Sox2+, MCM2+) and type 2b/proliferating type 3 cells (GFP+, GFAP−, Sox2−, MCM2+) at 4 wk post tamoxifen injection. A trend toward statistical significance ($P = 0.068$) was observed for increased type 2a cells in $K_v1.1$ cKO mice ($n = 7$) compared to $K_v1.1$ WT mice ($n = 5$) at 4 wk post tamoxifen injection. No difference was noted in the amount of type 2b/3 cells between the two genotypes ($P = 0.84$). (*D*) Representative images of type 2a and type 2b/3 cells of $K_v1.1$ cKO mice and $K_v1.1$ WT mice at 4 wk post tamoxifen injection are shown with merged and individual expression of GFP (red), GFAP (blue), Sox2 (cyan), and MCM2 (green). (Scale bar, 10 µm.) (*E* and *F*) Quantification (cells per cubic millimeter) of type 2a (GFP+, GFAP−, Sox2+, MCM2+) and type 2b/3 cells (GFP+, GFAP−, Sox2−, MCM2+) at 8 wk post tamoxifen injection. We were unable to determine with confidence if type 2a (GFP+, GFAP−, Sox2+, MCM2+) cell counts were altered in the $K_v1.1$ cKO mice at 8 wk post tamoxifen injection ($P = 0.14$). We did not find a difference in type 2b/3 cells (GFP+, GFAP−, Sox2−, MCM2+) ($P = 0.27$) between $K_v1.1$ cKO mice ($n = 6$) and $K_v1.1$ WT mice ($n = 4$). (*G*) Representative image of type 2a and type 2b/3 cells

in $K_v1.1$ cKO mice and $K_v1.1$ WT mice at 8 wk post tamoxifen injection are shown with merged and individual expression of GFP (red), GFAP (blue), Sox2 (cyan), and MCM2 (green). (Scale bar, 10 μm.) (*B*, *C*, *E*, and *F*) Unpaired two-tailed Student's *t* test. $P < 0.10$ indicated. Data are presented as mean $\pm$ SEM.

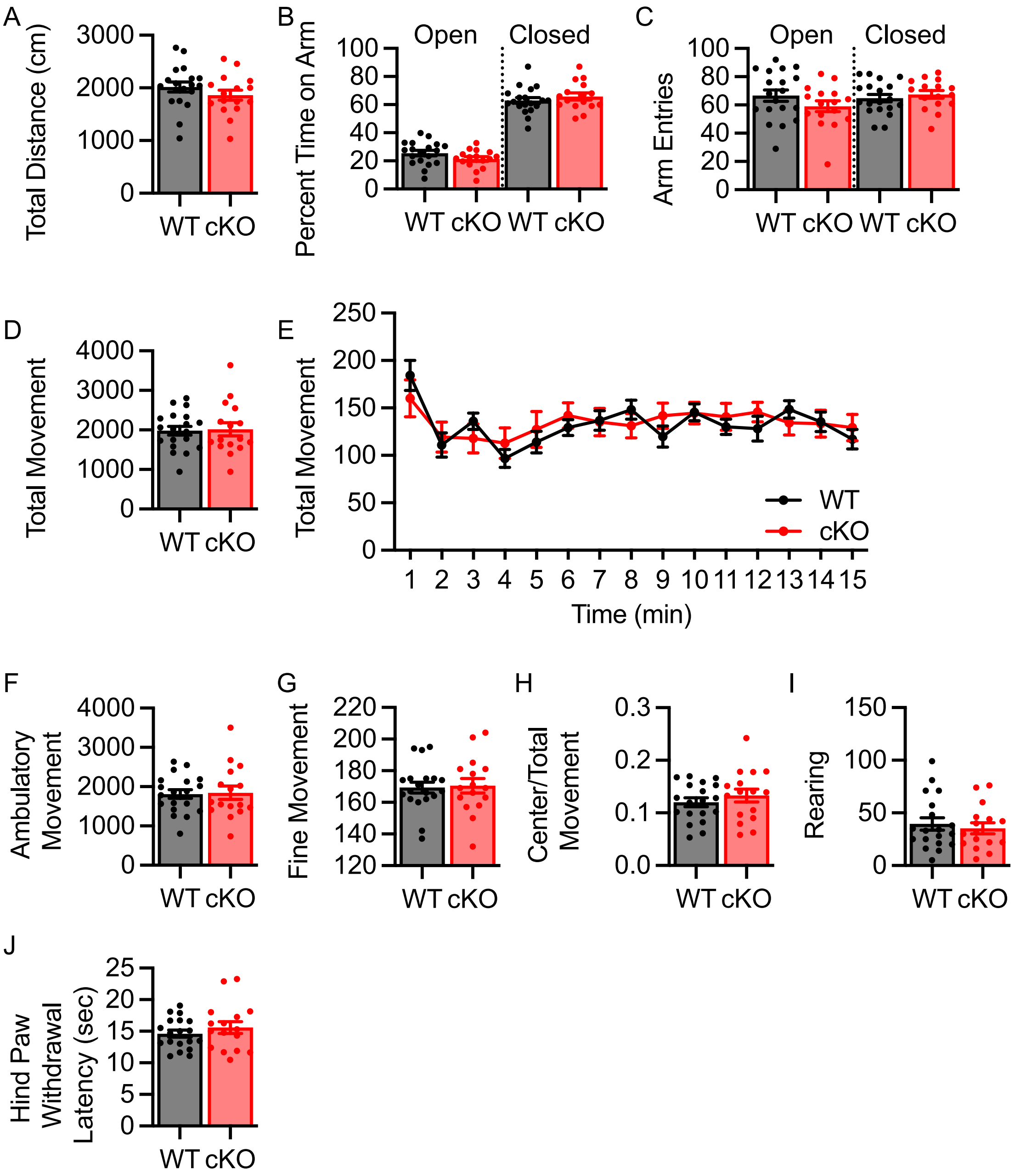

Fig. S5. K_v1.1 cKO mice show no abnormalities in elevated plus maze, open field, and hot plate test. (*A*–*C*) Charts displaying behavior of K_v1.1 cKO (*n* = 16) and K_v1.1 WT mice (*n* = 19) on the elevated plus maze. No significant differences were found in locomotor activity (total distance traveled [*P* = 0.27]), exploration of the open arms (percent time on open arm [*P* = 0.11], percent time on closed arm [*P* = 0.38], open arm entries [*P* = 0.18], or closed arm entries [*P* = 0.49]). (*D*–*I*) Charts showing behavior of K_v1.1 cKO and K_v1.1 WT mice on the open field test. No significant differences were seen in locomotor activity (total movement [*P* = 0.86], total movement over time between genotype [*P* = 0.86], ambulatory movement [*P* = 0.87], fine movement [*P* = 0.84]), baseline anxiety (center/total movement [*P* = 0.40]), or exploratory activity (rearing [*P* = 0.79]). (*J*) Quantification comparing pain sensitivity of K_v1.1 cKO and K_v1.1 WT mice via hotplate test. No difference in pain sensitivity, as measured by hind paw withdrawal latency (*P* = 0.38) was observed. (*A*, *D*, *F*, *G*, and *J*) Unpaired two-tailed Student's *t* test with Welch's correction; (*B* and *C*) Unpaired two-tailed Student's *t* test with Welch's correction comparing genotypes for both open and closed

arms; (*E*) Linear mixed-model with restricted maximum likelihood (REML) and Geisser-Greenhouse correction; (*I*) Unpaired two-tailed Mann Whitney *U* test. Data are presented as mean $\pm$ SEM.

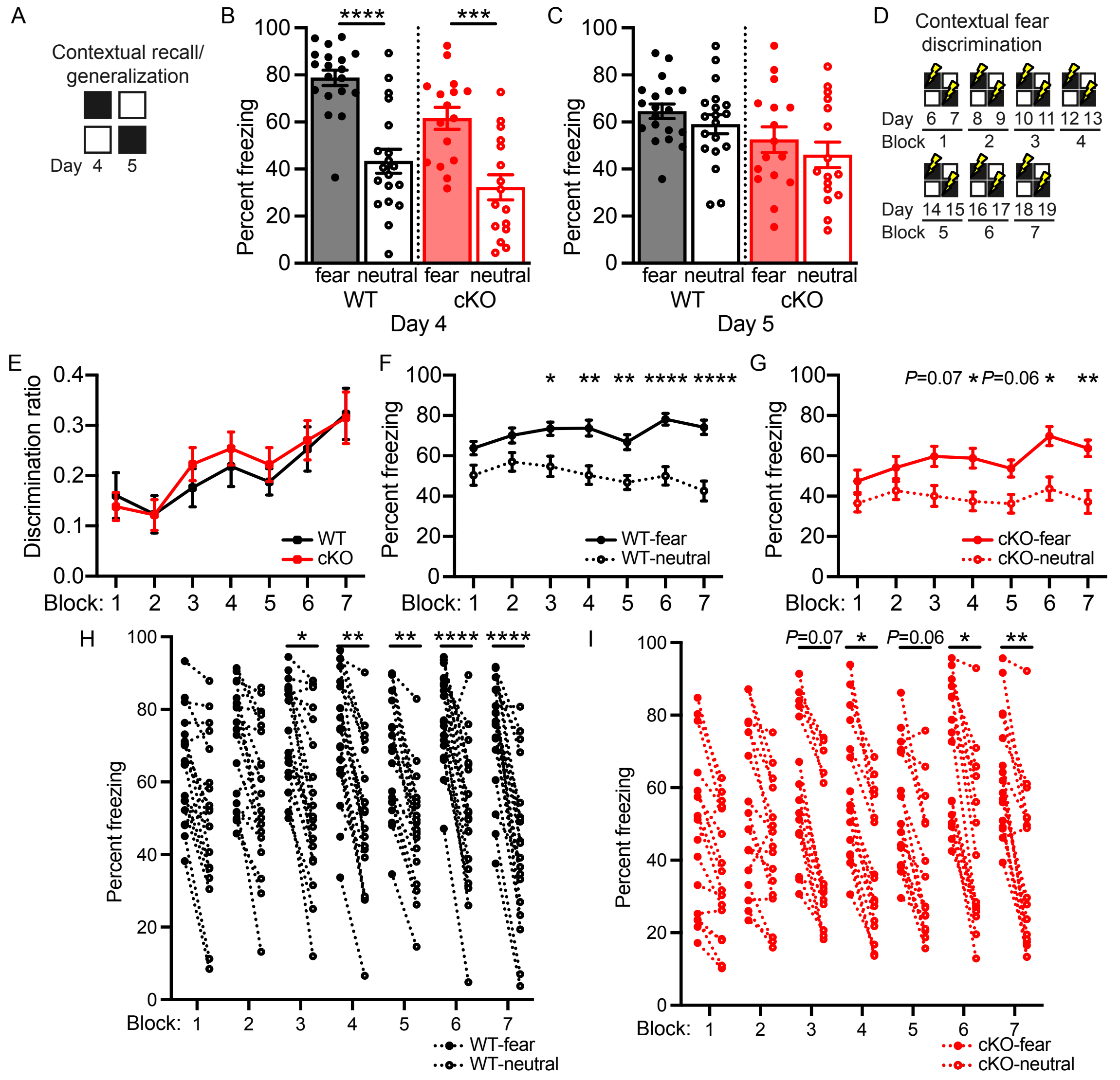

Fig. S6. K_v1.1 cKO mice show no impairments in contextual fear generalization and contextual discrimination, as compared to K_v1.1 WT mice. (*A*) Protocol for contextual recall/generalization. After 3 d of contextual fear conditioning, K_v1.1 cKO (n = 16) and K_v1.1 WT (n = 19) mice were exposed to the fear context (indicated by a black box) without shock to test their recall in the morning and introduced to a new shock-free neutral context (indicated by a white box) to test their generalization in the afternoon of day 4. On day 5, the order of the two contexts was switched. (*B* and *C*) Quantification of percent freezing in the fear and neutral context on day 4 and 5. On day 4, both genotypes froze more in the fear context than the neutral context (K_v1.1 WT [$P < 0.0001$], K_v1.1 cKO [$P = 0.0003$]). As the mice were not shocked in either context on day 4, they experienced an extinction of the freezing response in the fear context and generalized their freezing responses to the neutral context, resulting in a similar percent freezing in both contexts within genotype on day 5 (K_v1.1 WT [$P = 0.28$], K_v1.1 cKO [$P = 0.41$]). Unpaired two-tailed Student's t test with Welch's correction. (*D*) Protocol for contextual discrimination with shock administered only in the fear context (indicated by a black box with shock symbol). Data from 2 d were averaged to form a block. (*E*) Chart comparing the discrimination ratio ([percent freezing in fear context – percent freezing in neutral context] / [percent freezing in fear context + percent freezing in neutral context]) of K_v1.1 cKO and K_v1.1 WT mice over time. The two genotypes showed no difference in their discrimination ratios. Linear mixed-model with REML and Geisser-Greenhouse correction followed by Sidak's

multiple comparisons test: genotype effect ($F_{1, 33} = 0.11$, $P = 0.74$), block effect ($F_{4.3, 142} = 14$, $P < 0.0001$), and genotype × block interaction ($F_{6, 198} = 0.51$, $P = 0.80$); Sidak's multiple comparisons: block 1 ($P > 0.99$), block 2 ($P > 0.99$), block 3 ($P = 0.95$), block 4 ($P > 0.99$), block 5 ($P = 0.98$), block 6 ($P > 0.99$), and block 7 ($P > 0.99$). (*F* and *G*) Chart of percent freezing in the two contexts within each genotype over time, showing similar trends between $K_v1.1$ cKO and $K_v1.1$ WT mice. Linear mixed-model with REML and Geisser-Greenhouse correction followed by Sidak's multiple comparisons test between fear and neutral context for both genotypes; $K_v1.1$ WT: context effect ($F_{1, 36} = 17$, $P = 0.0002$), block effect ($F_{3.7, 133} = 4.8$, $P = 0.0017$), and context × block interaction ($F_{6, 216} = 5.2$, $P < 0.0001$); Sidak's multiple comparisons: block 1 ($P = 0.20$), block 2 ($P = 0.21$), block 3 ($P = 0.028$), block 4 ($P = 0.0036$), block 5 ($P = 0.0028$), block 6 ($P < 0.0001$), and block 7 ($P < 0.0001$); $K_v1.1$ cKO: context effect ($F_{1, 30} = 8.8$, $P = 0.0058$), block effect ($F_{3.5, 104} = 9.6$, $P < 0.0001$), and context × block interaction ($F_{6, 180} = 4.4$, $P = 0.0003$); Sidak's multiple comparisons: block 1 ($P = 0.64$), block 2 ($P = 0.59$), block 3 ($P = 0.070$), block 4 ($P = 0.023$), block 5 ($P = 0.061$), block 6 ($P = 0.011$), and block 7 ($P = 0.0055$). (*H* and *I*) Charts showing individual data points of *SI Appendix*, Fig. S6 *F-G* with percent freezing of individual mice in the two contexts connected by a dotted line within each block. $P < 0.10$ indicated, * $P < 0.05$, ** $P < 0.01$, *** $P < 0.001$, **** $P < 0.0001$. Data are presented as mean ± SEM.

SI References

1. K. J. Livak, T. D. Schmittgen, Analysis of relative gene expression data using real-time quantitative PCR and the $2^{-\Delta\Delta C_T}$ Method. *Methods* **25**, 402-408 (2001).
2. B. Robertson, D. Owen, J. Stow, C. Butler, C. Newland, Novel effects of dendrotoxin homologues on subtypes of mammalian K_v1 potassium channels expressed in Xenopus oocytes. *FEBS Lett* **383**, 26-30 (1996).
3. J. A. Verheugen, H. P. Vijverberg, M. Oortgiesen, M. D. Cahalan, Voltage-gated and Ca^{2+}-activated K^+ channels in intact human T lymphocytes. Noninvasive measurements of membrane currents, membrane potential, and intracellular calcium. *J Gen Physiol* **105**, 765-794 (1995).
4. J. A. Verheugen, D. Fricker, R. Miles, Noninvasive measurements of the membrane potential and GABAergic action in hippocampal interneurons. *J Neurosci* **19**, 2546-2555 (1999).
5. T. Nakashiba *et al.*, Young dentate granule cells mediate pattern separation, whereas old granule cells facilitate pattern completion. *Cell* **149**, 188-201 (2012).
6. T. E. Tracy *et al.*, Acetylated Tau Obstructs KIBRA-Mediated Signaling in Synaptic Plasticity and Promotes Tauopathy-Related Memory Loss. *Neuron* **90**, 245-260 (2016).
